# Unraveling Extremely Damaging *IRAK4* Variants and Their Potential Implications for IRAK4 Inhibitor Efficacy

**DOI:** 10.3390/jpm13121648

**Published:** 2023-11-26

**Authors:** Mohammed Y. Behairy, Refaat A. Eid, Hassan M. Otifi, Heitham M. Mohammed, Mohammed A. Alshehri, Ashwag Asiri, Majed Aldehri, Mohamed Samir A. Zaki, Khaled M. Darwish, Sameh S. Elhady, Nahla H. El-Shaer, Muhammad Alaa Eldeen

**Affiliations:** 1 Department of Microbiology and Immunology, Faculty of Pharmacy, University of Sadat City, Sadat City 32897, Egypt; mohamedyehya950@gmail.com; 2 Department of Pathology, College of Medicine, King Khalid University, Abha P.O. Box 61421, Saudi Arabia; raeid@kku.edu.sa (R.A.E.); hotifi@kku.edu.sa (H.M.O.); 3 Department of Anatomy, College of Medicine, King Khalid University, Abha P.O. Box 61421, Saudi Arabia; mutwakilheitham@gmail.com (H.M.M.); maldehri@kku.edu.sa (M.A.); mszaki@kku.edu.sa (M.S.A.Z.); 4 Department of Child Health, College of Medicine, King Khalid University, Abha P.O. Box 62529, Saudi Arabia; mohamed8964@hotmail.com (M.A.A.);; 5 Department of Medicinal Chemistry, Faculty of Pharmacy, Suez Canal University, Ismailia 41522, Egypt; khaled_darwish@pharm.suez.edu.eg; 6 Department of Natural Products, Faculty of Pharmacy, King Abdulaziz University, Jeddah 21589, Saudi Arabia; sselhady83@gmail.com; 7 Department of Zoology, Faculty of Science, Zagazig University, Zagazig 44511, Egypt; nhelshaer@zu.edu.eg

**Keywords:** innate immunity, SNPs, bioinformatics, molecular dynamics, TLR signaling

## Abstract

Interleukin-1-receptor-associated kinase 4 (IRAK4) possesses a crucial function in the toll-like receptor (TLR) signaling pathway, and the dysfunction of this molecule could lead to various infectious and immune-related diseases in addition to cancers. *IRAK4* genetic variants have been linked to various types of diseases. Therefore, we conducted a comprehensive analysis to recognize the missense variants with the most damaging impacts on IRAK4 with the employment of diverse bioinformatics tools to study single-nucleotide polymorphisms’ effects on function, stability, secondary structures, and 3D structure. The residues’ location on the protein domain and their conservation status were investigated as well. Moreover, docking tools along with structural biology were engaged in analyzing the SNPs’ effects on one of the developed IRAK4 inhibitors. By analyzing *IRAK4* gene SNPs, the analysis distinguished ten variants as the most detrimental missense variants. All variants were situated in highly conserved positions on an important protein domain. L318S and L318F mutations were linked to changes in IRAK4 secondary structures. Eight SNPs were revealed to have a decreasing effect on the stability of IRAK4 via both I-Mutant 2.0 and Mu-Pro tools, while Mu-Pro tool identified a decreasing effect for the G198E SNP. In addition, detrimental effects on the 3D structure of IRAK4 were also discovered for the selected variants. Molecular modeling studies highlighted the detrimental impact of these identified SNP mutant residues on the druggability of the IRAK4 ATP-binding site towards the known target inhibitor, HG-12-6, as compared to the native protein. The loss of important ligand residue-wise contacts, altered protein global flexibility, increased steric clashes, and even electronic penalties at the ligand–binding site interfaces were all suggested to be associated with SNP models for hampering the HG-12-6 affinity towards IRAK4 target protein. This given model lays the foundation for the better prediction of various disorders relevant to IRAK4 malfunction and sheds light on the impact of deleterious IRAK4 variants on IRAK4 inhibitor efficacy.

## 1. Introduction

IRAK4 is a constituent of the toll-like receptor signaling pathway, which assumes a crucial function, particularly in the context of innate immunity [1]. The researchers generated knockout mice lacking IRAK4 and observed that these animals exhibited significant impairments in their immune responses to viral and bacterial challenges. This finding highlighted the crucial involvement of IRAK4 in the signaling pathways of toll-like receptors (TLRs) and interleukin-1 receptors (IL-1Rs) [2,3]. IRAK4, a serine–threonine kinase, engages in interaction with MyD88, followed by the phosphorylation of downstream molecules. This cascade ultimately culminates in the activation and translocation of nuclear factor-κB (NF-κB) to the nucleus, leading to the transcription of inflammatory mediators, including IL-6 [4]. It has been found that the clustering of IRAK4 molecules leads to the activation of kinase activity, which causes IRAK1 to be phosphorylated, ubiquitinated, and degraded, which stimulates nuclear factor κB (NF-κB) and downstream mitogen-activated protein kinase (MAPK) [5]. IRAK4 malfunction plays an important role in the initiation of different immune-related diseases and cancers [6].

Owing to its role in TLR signaling, IRAK4 is involved in the release of several inflammatory cytokines and chemokines; therefore, hyperactivity of IRAK4 signaling can ultimately lead to several inflammatory disorders such as rheumatoid arthritis and inflammatory bowel disease [7]. Additionally, it was reported that cases with melanin cell tumors experienced an elevated state of IRAK4 phosphorylation, where IRAK4 knockdown resulted in tumor growth inhibition [8]. Moreover, the overstimulation of IRAK4, which activates the innate immune responses and the inflammatory cascade, can ultimately lead to the onset of prostate cancer [9]. Matching with these findings, a direction of IRAK4 inhibitor design was developed, especially after confirming that in later stages of life, IRAK4 inhibition has promising results against different diseases while maintaining immunity [10].

A missense mutation refers to a specific category of SNP that leads to alterations in the sequence of amino acid residues during translation [11]. The occurrence of a missense mutation has the potential to result in the synthesis of a modified protein with alterations in both its structure and function, thereby contributing to the development of various diseases. The identification of these specific single-nucleotide polymorphisms (SNPs) that are responsible for pathogenic effects continues to represent a challenge for researchers in the field [12]. Moreover, studying the association between genetic variants and different disorders has received significant attention in recent years [13,14,15]. Given the extensive reporting and deposition of missense single-nucleotide polymorphisms (SNPs) in databases, it has become imperative to employ a filtration process to distinguish SNPs with potential pathogenicity from the larger pool of neutral variants [16,17].

Despite the accuracy and reliability of experimental techniques when evaluating the implications of a substitution, the elevated cost, time consumption, and complexity associated with the experimental procedures when analyzing the whole single-nucleotide polymorphisms (SNPs) within the genome or even within a single gene pose an obstacle for researchers [18]. Computational methodologies have been extensively employed as a viable option to evaluate the potential consequences of amino acid residue alterations in proteins, aiming to determine their deleterious nature [19,20]. This has accompanied the recent remarkable usage of computational methods in initial studies in many biological fields [21,22]. Research has indicated that the presence of *IRAK4* genetic variants is associated with various infectious diseases, including severe sepsis, heightened occurrence of Gram-positive infection, and increased severity and vulnerability of enterovirus 71 infection [2,23,24]. Furthermore, there is evidence suggesting a correlation between genetic variations in *IRAK4* and the occurrence of certain types of cancer, such as breast cancer, as well as the advancement of hepatocellular carcinoma (HCC) related to hepatitis B virus (HBV) [25,26]. Therefore, uncovering *IRAK4’s* most deleterious variants would enable better monitoring of the liability of these disorders.

In addition, several pharmaceutical companies are currently testing different small-molecule inhibitors of IRAK4 as potential drugs against rheumatoid arthritis, inflammatory bowel disease, and hematologic malignancies [27]. Despite the current progress in IRAK4 inhibitor development, it is still unknown whether *IRAK4* SNPs would interfere with the binding between IRAK4 and its designed inhibitors. From all the above, the necessity was felt for conducting a comprehensive in silico study of *IRAK4* missense SNPs to pinpoint the most damaging variants. In addition, docking tools along with structural biology were employed to analyze the effect of these filtered variants on the binding between IRAK4 and its designed inhibitors by studying this relationship with HG-12-6. It is anticipated that this development will enhance the prominence of personalized medicine and facilitate the enhancement of management and protective protocols for many relevant disorders.

## 2. Results

### 2.1. General Information

**Figure 1:**
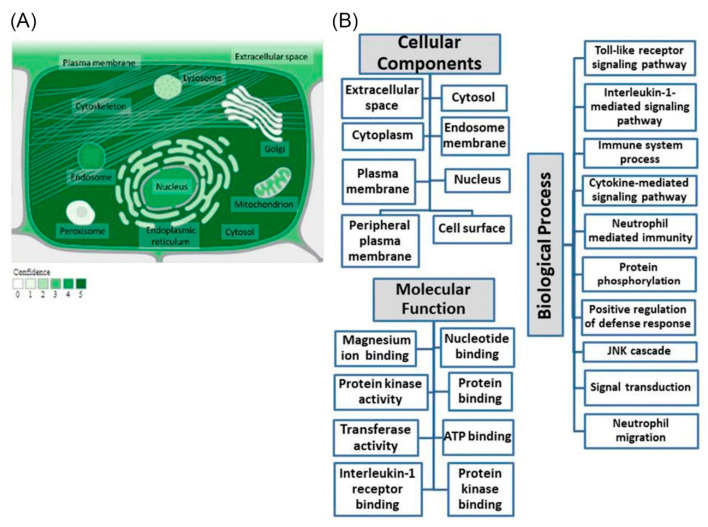
(**A**) *IRAK4* subcellular localization. Color code signifies the confidence level, with a range of colors from light green, which signifies a low level, to dark green, which signifies a high level (genecards.org; accessed on 11 June 2023), with the image’s origin being compartments.jensenlab.org. (**B**) Investigation of *IRAK4’s* gene ontology. Terms of biological process, molecular function, and cellular components regarding *IRAK4* are revealed (genecards.org; accessed on 20 July 2023).

The *IRAK4* gene signifies a protein-coding gene assigned to the NCBI Gene ID 51135. The genomic locus of the *IRAK4* gene is situated at 12q12 and comprises fifteen exons, spanning a total length of 30,591 nucleotides. Ensemble.org reports the existence of eighteen transcripts for the *IRAK4* gene. The subcellular localization regarding the *IRAK4* gene is shown in Figure 1A (compartments.jensenlab.org; accessed on 11 June 2023). Additionally, Figure 1B displays the gene ontology of *IRAK4* (genecards.org; accessed on 20 July 2023).

### 2.2. IRAK4 Gene Variant Retrieval

Retrieving *IRAK4* gene SNPs (accessed on 9 March 2023) revealed the presence of 388 missense SNPs, 139 synonymous variants, 9253 introns, 913 5′ untranslated region (UTR), and 963 3′ UTR SNPs, along with various other variants located upstream as well as downstream.

### 2.3. Predicting Variants That Exhibit the Most Deleterious Implications

The study employed six diverse bioinformatics tools, namely SIFT, PolyPhen-2, SNAP2, PANTHER, SNP&GO, and PHD-SNP, to pinpoint deleterious variants in the *IRAK4* gene that exert significant effects on the protein function of IRAK4. Out of the total 388 missense SNPs, a subset of ten missense SNPs was identified as deleterious by the six utilized tools. Table 1 displays the outcomes and evaluations of the ten SNPs utilizing distinct tools. 

### 2.4. The Examination of the Impact of Genetic Variations on the Stability of IRAK4 Protein

The present study evaluated the impact of specific variants on the stability of IRAK4, utilizing the I-Mutant 2.0 along with Mu-Pro computational tools. Table 2 presents the relevant values in conjunction with the conclusions and findings of the analysis. The stability of IRAK4 was observed to decrease due to the presence of eight SNPs by both tools, while G198E was found by Mu-Pro to reduce IRAK4 stability. All these nine damaging SNPs were subjected to further analysis.

### 2.5. The Identification of the Positioning of SNPs on IRAK4 Protein Domains

The application of InterPro demonstrated the presence of two domains known as the protein kinase (InterPro accession: IPR000719) and the death domain (InterPro accession: IPR037970). Table 3 displays the identification of all the selected variants within the protein kinase domain, as indicated by the analysis.

### 2.6. Secondary Structure Analysis

The PSIPRED technique was employed to predict the IRAK4 secondary structure, as depicted in Figure S1A. Table 3 presents the findings of the analysis conducted on the chosen positions in the wild form. In addition, IRAK4 secondary structures were studied in the case of the specified mutations, as demonstrated in Table 3. The mutations L318S and L318F correlated with variations in the secondary structures of IRAK4. The wild form’s coil structure was expected to be altered to a strand structure in the mutated forms, as depicted in Figure S1B and Figure S1C, respectively.

### 2.7. The Examination of the Conservation of IRAK4 Protein Residues in Terms of Phylogenetics

The ConSurf tool was employed to conduct a phylogenetic conservation analysis regarding IRAK4 protein residues (Figure S2). The study revealed that all SNPs were situated in regions of high conservation. The detailed findings and corresponding scores are presented in Table 3.

### 2.8. Conducting an Analysis of the Impact of the Determined Variants on IRAK4 Protein Structure

**Figure 2:**
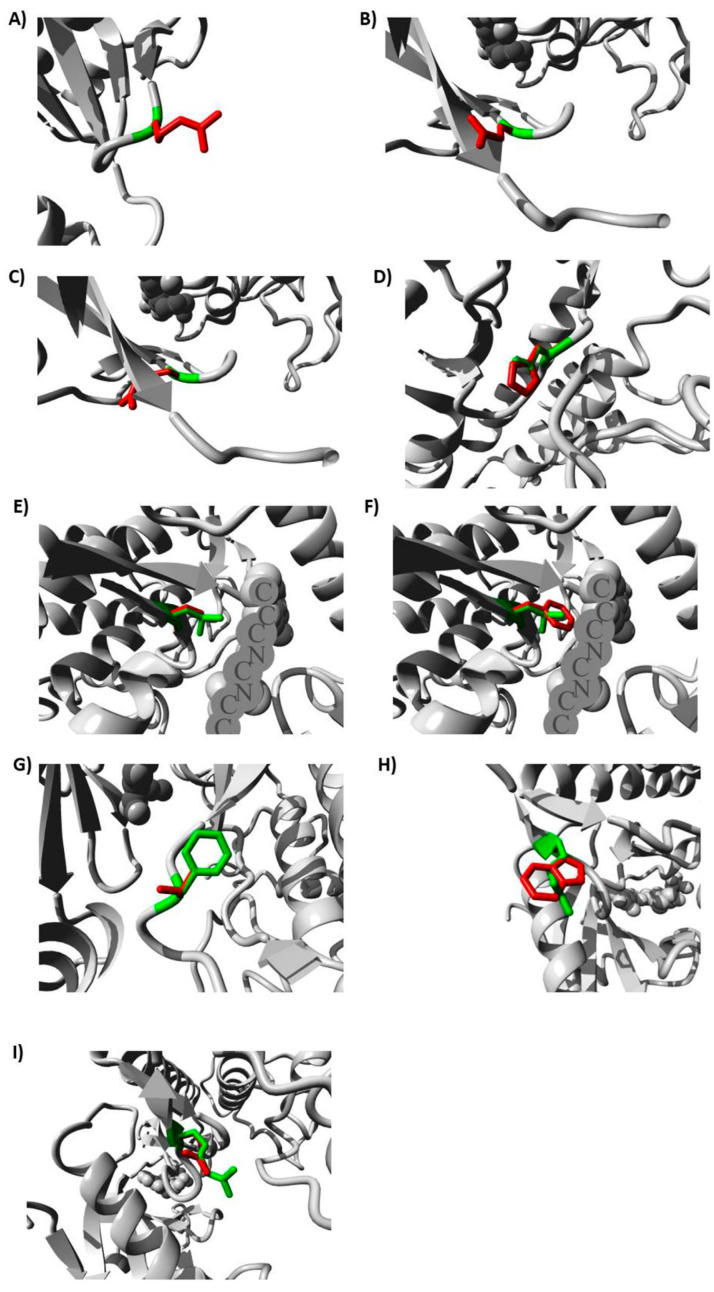
Effects of the studied variants on IRAK4 3D structure produced by HOPE server: (**A**) with G195R, (**B**) with G198E, (**C**) with G198R, (**D**) with D311H, (**E**) with L318S, (**F**) with L318F, (**G**) with F330V, (**H**) with R334W, and (**I**) with R334Q.

The investigation was expanded to examine the most deleterious variants’ impact on the IRAK4 protein structure through the utilization of the HOPE tool. Table 4 provides a detailed description of the structural consequences resulting from the substitution of amino acids in the IRAK4 protein. Figure 2 illustrates the substitution of the wild-type residue with the mutant counterparts with the most damaging SNPs.

### 2.9. Gene–Gene Interactions Analysis

**Figure 3:**
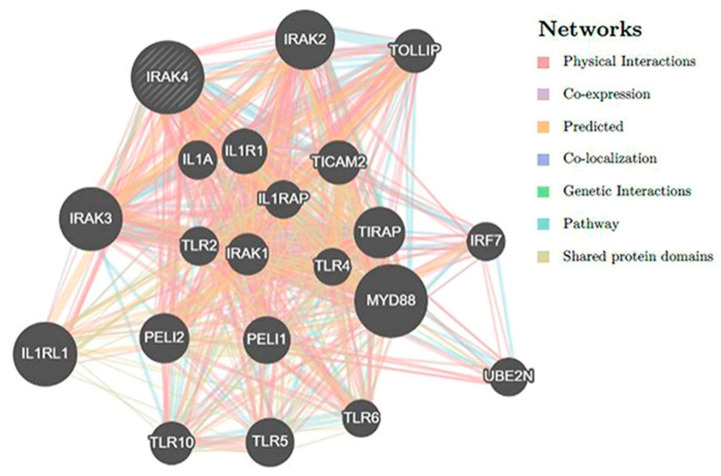
A network of *IRAK4* gene–gene interactions produced by GeneMANIA.

GeneMANIA was employed for producing the network related to *IRAK4* gene–gene interactions to find genes that exhibit robust interactions with *IRAK4*. This analysis led to the determination of the 20 genes that are most closely connected to *IRAK4*, as depicted in Figure 3. The *MYD88* gene was found to possess the highest rank among the genes analyzed. Subsequently, the interleukin-1-receptor-like 1 (*IL1RL1*) attained the second position in the ranking, followed by the *IRAK3* gene.

### 2.10. Molecular Docking Analysis

**Figure 4:**
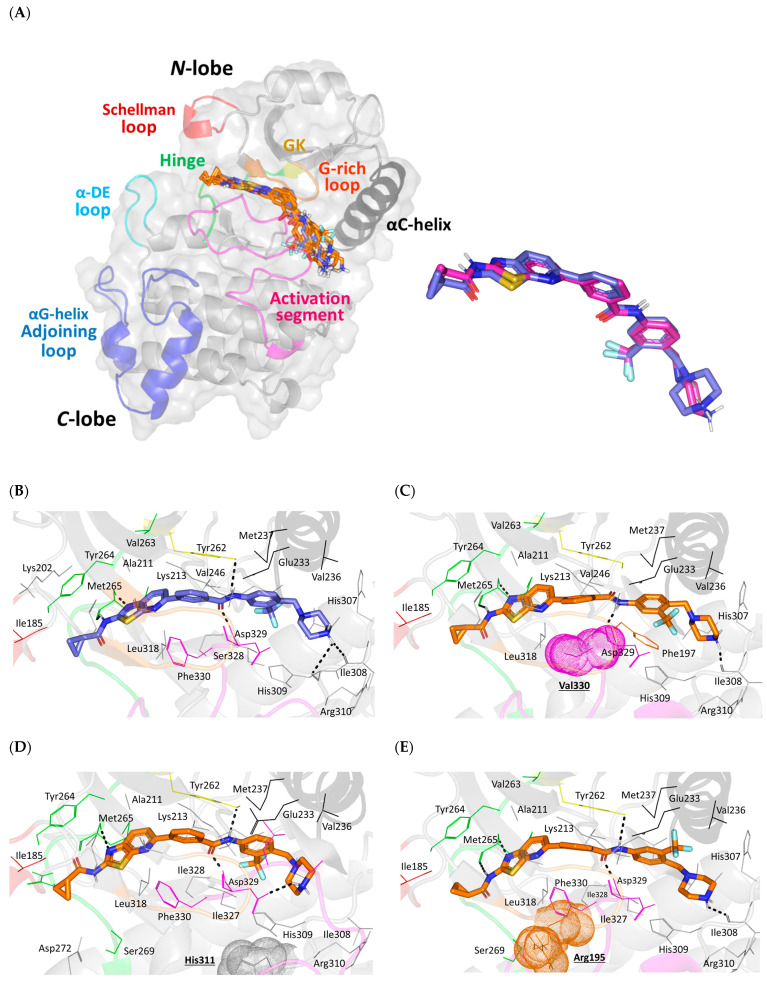

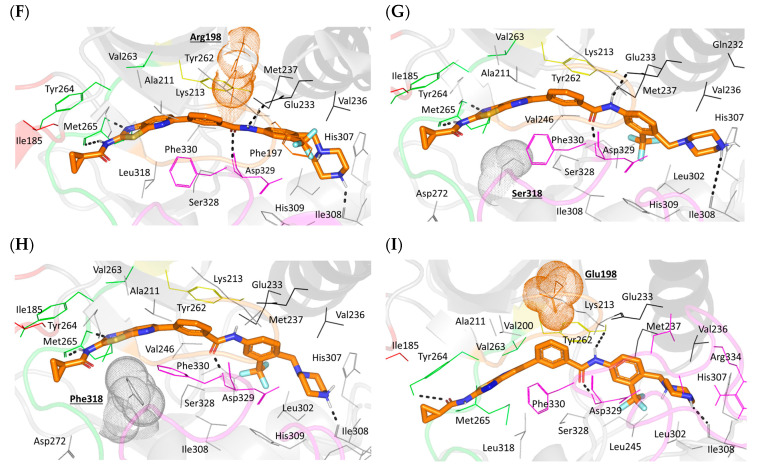
3D architecture and docking poses of HG-12-6 at IRAK4 native and SNP variants. (**A**) Overlaid binding modes of IRAK4/HG-12-6 models. The ligands are represented as blue sticks and orange lines at native and variant proteins, respectively. Native IRAK4 cartoon/surface is colored differently according to its structural motifs; hinge region (orange); Schellman loop (red), αDE loop (cyan); gatekeeper residue (GK; yellow), glycine-rich loop (orange); αC-helix (black); activation segment (magenta); and αG-helix/adjoining loops (blue). Right panel represents overlaid redocked and co-crystallized HG-12-6 at native IRAK4 for validating purposes of the docking protocol. (**B**–**I**) Binding modes of HG-12-6 at native IRAK4 (**B**) and its SNP mutants: (**C**) F330V; (**D**) D311H; (**E**) G195R; (**F**) G198R; (**G**) L318S; (**H**) L318F; and (**I**) G198E models. Residues located within 4 Å radius of bound ligand are displayed as lines, numbered with their protein sequence, and colored based on respective motif location. Only variant residues are in bold underlined text and shown with their surrounding electron densities in 3D dot representations. Polar interactions are shown as black dashed lines.

Molecular docking simulations for HG-12-6 at both the native and SNP variant proteins revealed adequate anchoring at the canonical ATP-binding site (Figure 4A). Docked ligands exhibited elongated conformation along the IRAK4-binding site with their terminal thiazolo [5,4-b]pyridine alkyl carboxamide scaffold being settled at the ATP–purine pocket close to the hinge region (Val263–Leu270) connecting both the *N*- and *C*-lobes of the target protein. This solvent-exposed pocket was endorsed by the down-directed *N*-lobe Schellman loop (Pro184–Gly189) and outward protruded *C*-lobe αDE loop (Cys276–Pro282) providing IRAK4′s distinctive extended surface area for the ATP-binding site. The latter provided shielded binding for the docked ligands’ thiazolo [5,4-b]pyridine alkyl carboxamide scaffold. On the other hand, the central benzamide core was settled near the IRAK4 designated gatekeeper residue (Tyr262) as well as being solvent-shielded via the *N*-lobe glycine-rich loop (GXGXXG; Gly193–Gly198). Beyond the gatekeeper residue, terminal benzyl piperazine moieties of the docked ligands were settled in the deep hydrophobic pockets, being mostly endorsed by the *N*-lobe αC-helix (Ile221–Cys240) and *C*-lobe activation segment (Asp329–Glu358). The distinctive DFG motif (Asp329–Phe330–Gly331) of the *C*-lobe activation segment was in close proximity to the ligand. Significant differential orientations/conformations of the ligand’s terminal benzyl piperazine moieties were depicted at SNP variant complexes in relation to the native IRAK4 protein. These depicted binding modes were in contrast to the poses at the hinge region, as quite superimposed conformations/orientations of the ligand’s terminal thiazolo [5,4-b]pyridine alkyl carboxamide scaffolds were depicted.

The ligand’s affinity towards the respective IRAK4 protein was translated via the estimated binding energies of the docked complexes being superior for HG-12-6 at native protein (−9.333 Kcal/mol), as compared to those of its variant isoforms (−7.667 Kcal/mol to −9.162 Kcal/mol) (Table 5). The latter was also interpreted into a low nanomolar affinity constant (*Ki*) for the native complex (147.014 nM) in relation to the SNPs complexes. Among the SNP IRAK4 complexes, the G198E protein had the most compromised binding affinity towards the ligand inhibitor (showing the least negative docking energy, −7.667 Kcal/mol), reaching its *K*i value up to a one-digit micromolar concentration. The L318F variant came in second place with a fair binding score (−8.313 Kcal/mol) and upper-range nanomolar affinity concentration (*K*i = 821.136 nM). On the contrary, the G195R, G198R, and L318S variants were only second to the native IRAK4 complex binding with moderate docking scores and affinity constants: −9.007 Kcal/mol 255.044 nM, −9.145 Kcal/mol 202.072 nM, and −9.162 Kcal/mol 196.292 nM, respectively. The latter could imply the higher detrimental impact of the negatively charged residue (glutamic acid) variant on HG-12-6 binding as compared to those of the positively charged (arginine) and even the small-sized polar amino acid (serine) variants. Differential binding affinity for the docked ligand at different protein isoforms could be further explained in terms of combined pocket residue-wise binding interactions towards the docked ligand inhibitor.

The comprehensive ligand residue binding analysis of the obtained poses revealed interesting findings. Regarding redocked HG-12-6 at the native IRAK4 protein, significant superimposition of the co-crystallized and redocked small molecule was depicted, showing a low RMSD value at 1.019 Å (Figure 4A). The redocked ligand managed to mediate double polar hydrogen bonding with Met265 at the hinge region as well as extended polar contacts via the ligand’s tail scaffold towards Tyr262, Ile308, His309, and Asp329 at the deep hydrophobic pockets (Figure 4B). Besides polar interactions, HG-12-6 was further stabilized via van der Waal hydrophobic contacts with the pocket’s non-polar residues (Ala211, Val236, Val246, Val263, Tyr264, Gly268, Leu302, Ile308, His309, Leu318, and Ile327). The ligand’s central benzamide core moiety was π-π sandwiched between the gatekeeper Tyr262 and Phe330 of the activation loop DFG motif, mediating stability for the anchored ligand. Interestingly, the Tyr262/Phe330-mediated π-π sandwiched anchoring was relevant for all ligand–SNP complex stability, except for F330V, since its variant residue lacked the essential aromatic character. The fair F330V binding energy was further highlighted by furnishing a low-extent hydrogen bonding network (just four hydrogen bonds with Met265, Ile308, and Asp329) (Figure 4C). Similar polar network data were depicted for the D311H model, where the ligand managed to mediate four hydrogen bonds, including only a single polar contact with the hinge region Met265 residue (Figure 4D).

Concerning the top-docked SNP variant complexes (G195R, G198R, and L318S), the conservation of native π-π sandwiched anchoring, besides furnishing five hydrogen bonding with pocket residues, was correlated to the relevant docking energies being only second to the native complex (Figure 4E–G). As compared to L318S, the bulkier residue variant L318F with its phenyl alanine amino acid was associated with higher steric clashes/hindrance than the small-sized residue variant analog Ser318 (Figure 4H). Another comparative variant analog showed the G-rich loop arginine variant residue at G195R and G198R to impose a possibly favored attractive positive/electronic microenvironment for the highly condensed π-electrons of the ligand’s aromatic core system. The latter would be translated into stabilized ligand anchoring, while contrarily, the glutamate variant analog G198E would cause ligand destabilization owing to its repulsive forces mediated by its inherited negative ionization. Notably, unfavored repulsive forces might explain why G198E was the only model where HG-12-6 mediated singular polar contact with Tyr264 instead of Met265 at the hinge region. The binding mode showed relative drift of the ligand’s terminal thiazolo [5,4-b]pyridine alkyl carboxamide scaffold towards the solvent-exposed entrance (Figure 4I)

### 2.11. Molecular Dynamics Simulation

**Figure 5:**
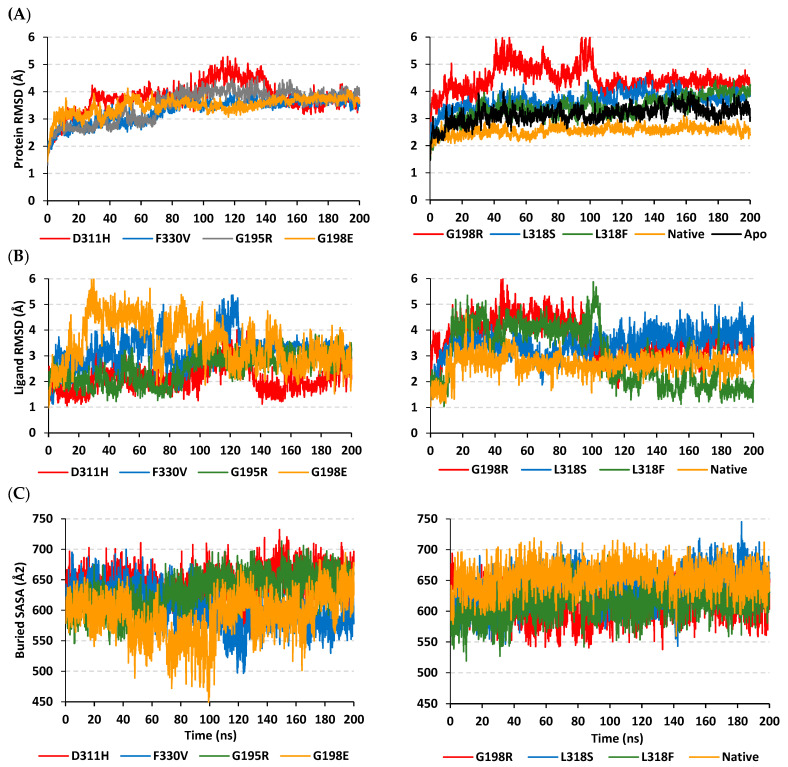
Analysis of molecular dynamics trajectories for HG-12-6-bound IRAK4 models. (**A**) Alpha-carbon RMSD of IRAK4 protein; (**B**) sole ligand’s RMSD; (**C**) buried SASA, plotted against the whole simulation timeline (200 ns).

The profound stability profile of the native IRAK4 complex over its SNP variants was highlighted through RMSD analysis of both the protein and sole ligand in reference to their corresponding initial structures. Simulated IRAK4 mutant proteins bound to HG-12-6 showed higher fluctuating tones and greater average alpha-carbon RMSD values (3.32 ± 0.43 Å to 4.456 ± 0.46 Å) as compared to those of the native protein (2.54 ± 0.17 Å) (Figure 5A). The highest fluctuating patterns (Up to 6.00 Å) were depicted for G198R from the simulation start until its half time (100 ns). Nevertheless, the G198R protein maintained its RMSD at a steady equilibrated plateau across the entire second half of the simulation timeframe. For the D311H variant protein, significant fluctuations were depicted from 100 to 140 ns, prior to leveling off beyond 140 ns until the end of the simulation. All the other SNP mutant IRAK4 proteins showed a gradual increase in their respective RMSD tones before they reached their own equilibration plateau for more than half of the simulation time (>140 ns). Similar patterns were observed for the apo and native IRAK4 proteins, yet both were seen at more steady trajectories and lower average RMSDs (3.16 ± 0.31 Å and 2.54 ± 0.17 Å, respectively), which was also most recognized for the native protein.

Regarding HG-12-6′s sole RMSD tones in relation to their reference/initial position, quite high fluctuations and RMSD values were depicted in the first 100 ns for the simulated ligands as compared to their respective bound mutant proteins (Figure 5B). The latter thermodynamic behavior was most prominent for the ligands at the SNP variants (3.98 ± 0.53 Å to 4.70 ± 0.89 Å) as compared to the native protein (2.70 ± 0.40 Å), conferring limited stability of earlier ligands at the pocket site. Notably, higher fluctuations were depicted for almost all ligands at the variant proteins in the first half of the simulation time before attaining a near-equilibrated state for the rest of the runs. Only HG-12-6 at G198E illustrated repeated fluctuation intervals across whole simulation.

Further investigation of the simulated models was highlighted through the calculated buried solvent-accessible surface area (B-SASA; Å^2^) for each simulated complex (Figure 5C). Interestingly, the B-SASA recapitulated the RMSD highlights regarding the detrimental impact of SNP mutations on IRAK4 global stability. The B-SASA corresponds to the amount of solvent-accessible surface area being buried within the interface between the ligand and its bound protein throughout the simulation. Using the equation developed by Zhang et al., the buried SASA was calculated through the equation B-SASA = 0.5 * (SASA_ligand_ + SASA_IRAK4_ − SASA_complex_) [28]. Depicting lower values in terms of B-SASA confers a respective reduced ligand–target interface denoting low buried surface area between both molecules. As expected, higher B-SASA tones were seen for the native IRAK4 complex (654.26 ± 22.58 Å^2^), while on the contrary, the SNP complexes were assigned lower BSA tones, with the least being for the simulated G198E model (591.08 ± 36.40 Å^2^). The latter SNP variant model was observed with non-steady tones as compared to all simulated systems.

**Figure 6:**
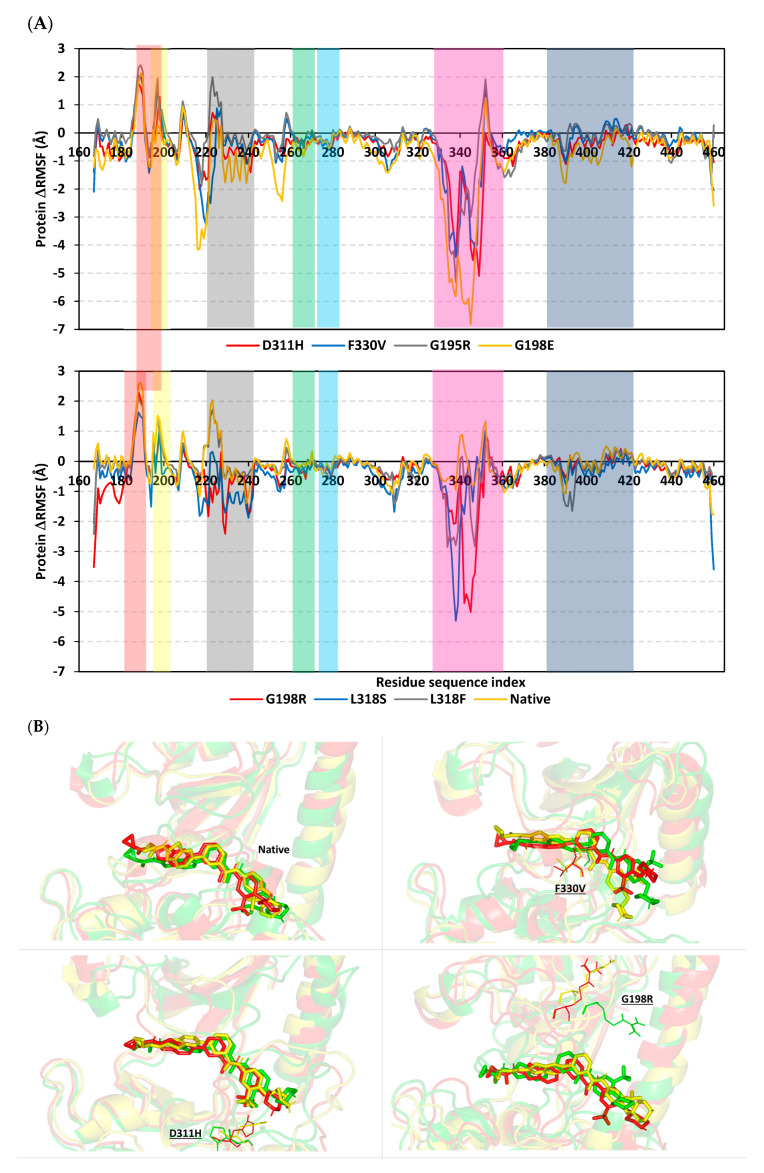

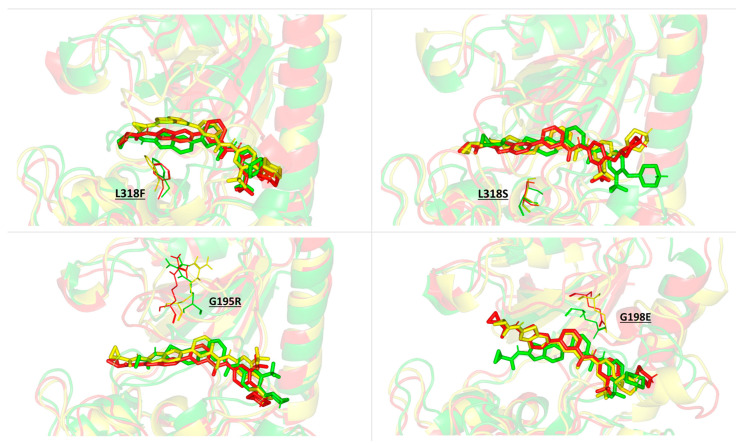
Global stability analysis for simulated HG-12-6-bound IRAK4 complexes. (**A**) Monitored ΔRMSF trajectories for simulated HG-12-6/IRAK4 models along the whole molecular dynamics simulations. Alpha-carbon differences in RMSF tones are illustrated in terms of constituting residue sequence number (Ser167 to Ser460). Important structure regions are highlighted in colors; hinge region (orange); Schellman loop (red), αDE loop (cyan); gatekeeper residue (GK; yellow), glycine-rich loop (orange); αC-helix (black); activation segment (magenta); and αG-helix/adjoining loops (blue). (**B**) Conformation evolution of the simulated complexes across the simulation timeline. Overlaid initial, middle, and final snapshots for native and SNP variant models at 0 ns, 100 ns, and 200 ns, respectively. Complexes are shown in green, yellow, and red cartoons with respect to the initial, middle, and final extracted frames. Ligands (sticks) and SNP residues (lines) are presented in colors corresponding to their extracted frames.

The stability profiles of the IRAK4 proteins, down to their secondary protein structure/motifs, as well as residue-wise levels, were evaluated by monitoring the protein’s alpha-carbon RMSF trajectories. Different RMSF values for a particular IRAK4 variant or wild-type model in relation to its respective apo state (ΔRMSF = RMSF_Apo_ − RMSF_Holo_) were represented for a better understanding of the protein’s residue-wise stability. Residues with ΔRMSF values ≥ 0.3 Å were considered significantly rigid, imposing negligible mobility, while ΔRMSFs below such a cut-off suggested higher protein flexibility patterns [29,30]. Notably, the IRAK4 structural motifs showed differential fluctuating/mobility profiles, almost at comparable trends across all the simulated models (Figure 6A). Both the Schellman and G-rich loops were assigned the top-immobile patterns, having the highest positive ΔRMSFs for their constituting residues. On the contrary, both the αC-helix and activation segments were depicted with high fluctuation patterns. However, the latter structural loop was assigned the highest negative ΔRMSFs as compared to any other IRAK4 secondary structure. Finally, the hinge region residue range and αDE loop showed moderate stability profiles across the simulated IRAK4 models.

Comparing the native IRAK4 protein with its mutant congregants revealed higher stability and rigidity patterns for the earlier protein state as being obvious across the whole residue range (Ser167 to Ser460). Less negative or more positive ΔRMSF tones were assigned to the native across every important structural motif/secondary structure of the IRAK4 protein. Notably, no significant fluctuation pattern was assigned to the αG-helix/adjoining loops. Focusing on the residue-wise stability of the native and SNP variant residues, G195R was assigned a negative ΔRMSF as compared to positive values for its wild-type residue (Supplementary Materials; Table S1). Regarding the SNP mutation for G198 at the G-rich loop, less positive ΔRMSFs were seen for the G198R and G198E protein, with a much lower number being assigned to the glutamate variant. Similar findings for the Asp318 variants were observed where more negative tones were depicted for L318S and L318F, with higher values for the latter variant. Interestingly, vicinal residues for the Asp318 variants were also assigned higher fluctuation patterns as compared to the other simulated congregants. Finally, the DFG motif at F330V depicted greater instability trends, being only second to the G198E variant protein.

The conformational analysis of the simulated models revealed quite a superimposition for the simulated HG-12-6 as well as the protein’s secondary structures at the native IRAK4 model across the initial (0 ns), middle (100 ns), and final (200 ns) timeframes (Figure 6B). On the contrary, significant ligand drift and/or protein structural torsions were depicted for the simulated SNP mutant complexes. Notably, the F330V model showed the loss of an adequate grip on the ligand’s central benzamide moiety, where the non-aromatic mutant valine residue depicted retraction and an increasing distance from the ligand site at 100 ns and 200 ns. Regarding both the D311H and G198R SNP variants, significant orientation or torsional alterations were observed for the respective Arg198 or His311 mutant residues at the middle and end of the simulation runs. These changes were associated with profound conformational movement/flexibility of the nearby protein’s secondary structures, including the activation loop, G-rich loop, and/or αC-helix. Moving to the L318F SNP model, the bulky mutant residue Phe318 was associated with the altered ligand’s orientation drifting its terminal thiazolo [5,4-b]pyridine alkyl carboxamide scaffold quite far from the hinge region site. On the other hand, the small-sized polar mutant residue at L318S depicted a significant drift of the whole ligand towards the pocket’s solvent side while losing its deep anchoring at the backside hydrophobic pockets, which was further associated with altered terminal phenyl piperazine conformations. Similar findings were seen with the ionized mutant residue of G195R where HG-12-6 was more solvent-exposed at the end of the simulation timeframe. Finally, the G198E model depicted the highest conformational and orientation changes for its respective simulated bound protein and ligand. The negatively ionized mutant residue was hampering near the ligand interface yet was associated with loss of the ligand’s hinge region contacts and highly altered conformations/orientations for the structural elements of the backside hydrophobic pocket.

**Figure 7:**
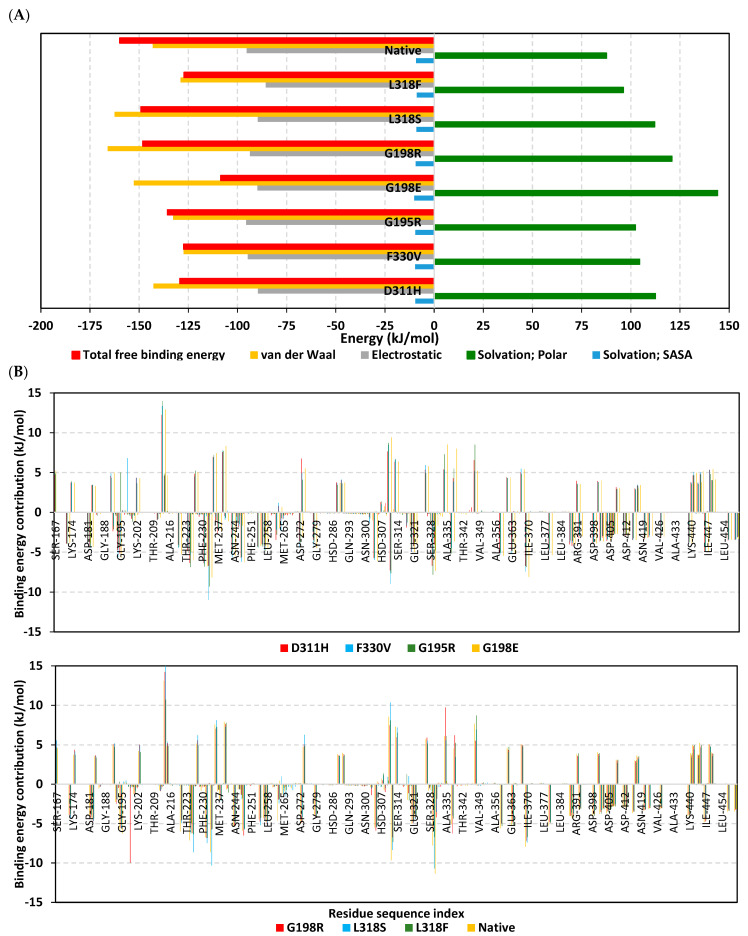
MM_PBSA free binding energy calculations for the HG-12-6-bound IRAK4 complexes. (**A**) Total free binding energies and their constituting energy terms. (**B**) Residue-based energy contributions.

The free binding energy of HG-12-6 towards simulated protein models was calculated using the trajectory-oriented MM-PBSA calculations, which predicted profound ligand’s affinity towards the native state (ΔG = −159.90 kJ/mol) (Figure 7A). The G198E model was assigned the lowest free binding energy (ΔG = −108.51 kJ/mol), while L318S was only second to the native model (ΔG = −149.06 kJ/mol), which was in agreement with our preliminary docking studies. Other G-rich SNP mutations had less compromised affinities towards HG-12-6 (ΔG = −135.62 kJ/mol for G195R and −148.14 kJ/mol for G198R). The binding energies at D311H, F330V, and L318F were of moderate values (ΔG ≈ −128.00 kJ/mol). Dissecting the furnished free binding energies into their energy term contributions illustrated dominant contributions, by almost two-fold, of the van der Waals potentials (ΔG van der Waals) over those of Coulomb’s electrostatic ones (ΔG electrostatics). Interestingly, hydrophobic energy contributions were higher at the SNPs G198E, G198R, and L318S, as compared to the native, while being comparable at D311H. The electrostatic contributions were quite similar across all IRAK4 models, including both SNP and the native state. Nevertheless, solvation penalties (ΔG solvation; polar) were higher for all SNPs in relation to the native model. It is worth mentioning that the non-polar solvation energy terms (ΔG solvation; SASA) were almost consistent for all simulated IRAK4 complexes, except for G198E and L318F, where values were higher and lower, respectively, in relation to the average values.

Decomposing the binding energies over the IRAK4-composing residues highlighted the key residue for attractive binding (high negative energy values) and those imposing unfavored repulsive forces against affinity (high positive energy values) (Figure 7B). Highly attractive residues were located at the G-rich loop, hinge region, and αDE loop. On the other hand, both the activation segment and αC-helix showed mixed energy contributions of both highly attractive and repulsive contact forces. Focusing on the reported key binding and SNP mutant residues, Gly198/x and Gly195/x showed negative attractive energy potentials for all the models except for SNP variants where positive repulsive unfavored forces were depicted. For the L318/x mutant variants, fair negative energy value contributions were depicted for L318S (−0.13 kJ/mol), whereas the bulky L318F congruent was at a positive repulsive energy value (1.24 kJ/mol). Regarding the Asp311 residue, high negative energy values were observed for several models, even for the D311H variant, despite the latter being of much lower contribution. Nonetheless, Asp311 at other SNP variants (G198/x and L318/x) were assigned high positive unfavored energy contributions (7.45 kJ/mol to 10.36 kJ/mol). Moving towards the gatekeeper residue, Tyr262 was furnished with favored energy contributions in all models (−2.14 kJ/mol to −4.64 kJ/mol). Exceptions were observed for the same four SNP variants observed with Asp311, where Tyr262 was assigned fair negative energy values (around −0.13 kJ/mol). Residue-wise energy contributions for the important DFG motif showed favored scores for both Asp329 and Phe330 over those of the Gly331 residue. However, these contributions were highly compromised for Val330, as expected in the F330V model, as well as for Asp329 in the G198/x and L318/x models.

## 3. Discussion

The essential involvement of IRAK4 in the innate canonical TLR pathway has been well established [31]. In addition, it has been established that IRAK4 deficiency is correlated with the compromise of toll-like receptor (TLR)/IL-1R-mediated immunity, resulting in heightened vulnerability to recurring bacterial infections that pose a significant risk to one’s life [32]. Furthermore, it has been observed that certain single-nucleotide polymorphisms (SNPs) in the *IRAK4* gene are correlated with various infectious and malignant diseases [2,23,24,25,26]. As a result, the identification of the most harmful missense single-nucleotide polymorphisms (SNPs) in the *IRAK4* gene has become imperative in the investigation process of this significant biomolecule.

A comprehensive analysis was conducted on *IRAK4* single-nucleotide polymorphisms. Among these, a subset of 388 SNPs were identified as missense variants and subsequently subjected to additional scrutiny. The mutations were subjected to analysis using six bioinformatics tools that employed diverse approaches and algorithms, thereby ensuring the robustness of the analysis. The collective assessment of these various tools identified a total of ten single-nucleotide polymorphisms that were predicted to possess deleterious effects and be implicated in disease. The analysis of the effects of these single-nucleotide polymorphisms on the stability of the IRAK4 protein was conducted using the I-Mutant 2.0 server and the Mu-Pro tool, as protein stability possesses a crucial role in determining protein function as well as its structure [33]. A total of eight single-nucleotide polymorphisms were identified through the utilization of I-Mutant 2.0 and Mu-Pro tools, which were observed to have a decreasing effect on the stability of IRAK4, while the G198E mutation was observed to have a decreasing effect with the Mu-Pro tool. All nine of these damaging SNPs were subjected to further analysis. After that, the protein underwent functional analysis in order to identify its crucial domains, utilizing the InterPro tool. The analysis exposed that all the selected variants were located in the protein kinase domain.

Additionally, considering the significance of a protein’s secondary structure and its crucial roles in the structure as well as the folding of the protein, the analysis of the secondary structure was carried out [34]. Using PSIPRED uncovered the existence of changes in the secondary structures of IRAK4 with L318S and L318F mutations. The ConSurf server was then used to examine the phylogenetic conservation, which revealed that all variants were located in highly conserved positions. Because residues of functional significance display high conservation scores [35], the presence of changes at these sites was anticipated to have functional impacts on the protein.

Furthermore, the nine detrimental SNPs were subjected to subsequent analysis utilizing the HOPE server. The aforementioned SNPs were anticipated to induce impairments in both the structure and functionality of the protein. The issue of engaging with gene–gene interactions gained significant importance in the investigation of disease–gene relationships, as it was confirmed that multiple genetic loci exhibit interactions among themselves [36]. The GeneMANIA tool was employed and identified that the MYD88 gene exhibited the most prominent interactions with the *IRAK4* gene, followed by the *IL1RL1* gene and *IRAK3* gene. The presence of *IRAK4* variants may potentially influence these genes that are closely linked to *IRAK4*.

In order to further investigate the impact of SNPs on IRAK4′s protein structure/function, a molecular modeling investigation, including molecular-docking-coupled dynamics simulations, was adopted to evaluate the druggability of IRAK4 towards known a potent inhibitor, HG-12-6. This thiazolo [5,4-b]pyridine-based chemical agent was introduced as an IRAK4 inhibitor with reported IC_50_ values of 2876.01 nM and 165.12 nM against the phosphorylated and unphosphorylated proteins, respectively, through the LanthaScreen-Eu^TM^ kinase-binding bioassay [37]. Molecular docking has been considered a significant in silico tool for predicting the respective ligand–target orientations and conformations within the target-binding sites [38]. This computational approach has been extensively applied within the literature for providing a differential binding analysis between its native and variant isoforms [17,39,40,41,42]. Notably, our adopted molecular docking protocol was confirmed to be highly valid since a low redocking RMSD value (0.947 Å) was obtained for the redocked co-crystalline ligand in relation to its reference orientation/conformation within the crystallized complex. Depicting RMSD values less than 2.0 Å signifies that both the adopted docking algorithms and parameters were efficient for predicting relevant binding poses that would ensure their respective biological significance and, in turn, the docking energies [43].

Findings within the presented molecular docking investigation illustrated that the seven IRAK4 SNP variants were hampered by lower binding affinities towards HG-12-6 as compared to the native protein. This could be rationalized since these variants’ residues were at IRAK4′s ATP-binding sites or adjoining pockets, which implies relevant impacts against HG-12-6 binding. Belonging to the kinase superfamily, IRAK4′s ATP-binding pocket is quite conserved across different kinase members [44,45,46]. A plethora of studies within the current literature have reported the detrimental impact of pocket residue SNP variants for kinase inhibitor binding, leading to significant drug resistance both at pre-clinical and clinical stages [47,48,49,50,51]. Typically, IRAK4′s pocket comprises a solvent-exposed hinge region and deep hydrophobic pockets with a gatekeeper residue guiding the entrance of the latter pockets [37,52,53]. Reported studies analyzing topological water networks at IRAK4′s ATP-binding site revealed crucial ligand binding towards the hinge region for achieving significant inhibition activity [6,54]. In these regards, our docked D311H and G198E complexes were the reasons for HG-12-6′s compromised binding energies and affinity constants since these variants would mediate limited or inappropriate ligand anchoring at the hinge region.

Regarding the deep hydrophobic pocket, a diverse class of inhibitors has probed this site for improving binding affinity as well as limiting the target’s promiscuity (improving ligand–target selectivity) [55]. The ability of ligand inhibitors to bind at these deep pockets would provide an advantage over conventional competition against IRAK4′s naturally highly abundant co-factor, ATP molecules [56]. Interestingly, most of the reported inhibitors targeting this backside pocket showed preference/selectivity for IRAK4′s unphosphorylated/inactive state, which was congruent with our adopted PDB file [57,58]. The accessibility of the backside hydrophobic pocket is typically mediated by the outward conformations of both the activation segment DFG motif and *N*-lobe αC-helix, being the hallmarks of the inactive IRAK4 state [57,58]. Reported analyses of topological water networks provided additional insights regarding this backside pocket being less congruent for reported IRAK4 inhibitors [6,54]. The latter suggested that binding at these hydrophobic sites would be at differential conformation/orientations, which was also highlighted within our docking investigation.

Flipping the Asp329 with Phe330 while keeping the Tyr262 gatekeeper in its place was reported to be inadequate for promiscuous kinase inhibitors where Phe330 provided ligand stability, while the gatekeeper residue hindered their entrance to IRAK4′s adjacent back pocket [37]. While 77% of human kinases are suited for large-sized gatekeepers (leucine, methionine, or phenylalanine) and 21% of tyrosine kinases for small ones (threonine or valine), the IRAK family members are characterized by tyrosine gatekeepers highlighting their substantial physiological significance [52]. All the above data would provide an explanation for F330V to depict a compromised docking score and affinity constant owing to its non-appropriate π-mediated stacking at the gatekeeper and DFG motif residues. Despite the great insights obtained from the docking simulation, clear explanations for the G195R, G198R, and L318 variant models are still in demand. It was suggested that altered protein flexibility, as well as solvation entropy due to these variant residues, would have a significant impact on IRAK4′s druggability and ligand–pocket accommodation. In these regards, the molecular dynamics simulations proceeded to grasp the thermodynamic behavior of all ligand–IRAK4 complexes at near-physiological conditions while accounting for solvation entropy, global protein flexibility, and free energy of binding.

The thermodynamic stability of the simulated models was tracked across the entire trajectories of the molecular dynamics runs through RMSD analysis. Typically, compromised stabilities and altered conformational profiles have long been correlated with high protein RMSDs, whereas ligands with excellent pocket accommodation/stability are correlated to steady/small-valued ligand RMSD tones [59]. It is worth noting that RMSD analysis further validated our molecular dynamics approach since the depicted RMSDs for all the simulated ligands never exceeded a two-fold difference in the values of their respective bound target proteins. This would confirm successful protein convergence and the adequacy of the prior minimization/equilibration stages requiring no further time extensions [60,61]. It is worth noting that both B-SASA and the differences in RMSF findings recapitulated the RMSD highlights regarding the detrimental impact of SNP mutations on IRAK4 global stability. Depicting lower values in terms of B-SASA confers a respective reduced ligand–target interface denoting a low buried surface area between both molecules, while higher values and ΔRMSFs suggest higher protein flexibility patterns upon SNP mutation.

Based on the ΔRMSF and conformational analysis, profound wild-type stability has been further highlighted in relation to SNP variants. Within the current literature, reported SNP analysis of different human targets has shown significant influence on ligand binding and target conformation owing to lower residue-wise binding contributions, compromised occupancies of contact distances, and/or steric clashes/hindrance with congruent protein interface [39,62,63,64]. The latter could explain the reduced protein stability and ligand binding affinity towards the IRAK4 SNP, imposing bulky mutant residues. Replacing the small-sized glycine in G195R, G198R, and G198E with bulkier-sized residues of arginine or glutamate would have increased the steric clashes towards the ligands and/or vicinal protein loops. These would have imposed significant momentum for altered ligand fitting and/or changes within the binding site topology/size. The current literature has highlighted the impact of G-rich loop mutations with increased drug resistances for ABL and ALK kinase member proteins against imatinib and crizotinib [65,66,67,68]. This was recapitulated within our study’s RMSF analysis, where higher fluctuations (more negative/less positive ΔRMSFs) were depicted for these mutant residues as compared to their wild-type congregants. Steric clashes could also be the reason for L318F mutant state introducing the bulkier phenylalanine instead of leucine being settled at ~5.80 Å from the ligand interface.

Besides the steric influence of bulky mutant residues, the impact on global protein stability/flexibility has also been highlighted via these residues within our ΔRMSF and conformational analyses. The increased mobility trajectories for bulky residues in the RMSF analysis were also associated with more flexible vicinal residues as compared to the wild-type protein. The higher-flexibility patterns were also extended to quite distant secondary structures and motifs of the IRAK4 protein, including the activation loop, the DFG motif, G-rich loop, αC-helix, as well as parts of the αDE and Schellman loops. Notably, several reported oncogenic mutations at different kinase family members revealed flexibility alterations within the proteins’ key regions participating in the kinase’s substrate recognition and activation [69,70,71]. This was also depicted across other protein families since mutations at human lysozyme proteins of the hydrolase enzymes revealed increased flexibility patterns being large, frequent, and with a long range of effects [72], all of which was in good agreement with our findings.

The interpretation of the above-described free binding energy analysis has further correlated the mutant residue impact on the IRAK4 protein flexibility both through steric and electrostatic parameters. The electronic effects of mutant residues at G195R, G198/x, and L318S were suggested to indirectly influence the ligand’s stability by imposing conformational changes on the pocket’s secondary structures. The latter was observed within our residue-wise MM_PBSA free binding energy contributions, where the mutant residue imposed repulsive energy forces that destabilized the vicinal, as well as causing quite distant residue ranges. This was in good agreement with our prior conformational analysis. The electronic impact of mutant residues was observed in several non-gatekeeper hotspots concerning drug resistances in mTOR kinase [73]. A study by Huang et al. provided computational evidence regarding mechanistic drug resistance for anaplastic lymphoma kinase mutations where they would influence both the target and drug energetic variabilities and structural transitions [74]. Another study on anaplastic lymphoma kinase showed that out-pocket mutations might induce a possible transferable chain of mutation effects based on MM_PB(GB)SA free binding energy computations [75]. Furthermore, kinase resistance against imatinib was correlated to a dynamic conformational shift for mutant targets and enthalpy/entropy balance for the activation loop and its DFG motif [76]. The latter was in good agreement with our MM_PBSA calculations, where higher polar solvation was correlated to compromised HG-12-6/pocket accommodation since binding is a solvent-substitution process. Moreover, our non-polar solvation calculations illustrated the impact of mutant residue sizes to influence the pocket topology/surface area, where F330V and L318F depicted lower and higher values, respectively. Finally, our MM_PBSA calculation was reported to be valid, highlighting the superior contributions of the van der Waal potentials as being correlated to the reported dominant hydrophobic nature of the IRAK4 pocket [6,37,52], as well as the binding sites of other kinase members [77,78,79,80,81,82,83].

## 4. Materials and Methods

### 4.1. General Information

The Ensemble database, along with the National Center for Biotechnology Information (NCBI) databases (https://www.ncbi.nlm.nih.gov/gene/51135; accessed on 9 March 2023), were employed to obtain comprehensive information pertaining to the gene encoding interleukin-1-receptor-associated kinase 4 (IRAK4). Furthermore, the database of genecards.org; accessed on 20 July 2023 was employed to obtain gene ontology information, whereas compartments.jensenlab.org represented a subcellular localization information source.

### 4.2. Retrieving the Genetic Variations in IRAK4 Gene

National Center for Biotechnology Information (NCBI) was employed for retrieving IRAK4 variants utilizing variation viewer along with selecting dbSNP as the source database (https://www.ncbi.nlm.nih.gov/variation/view/; accessed on 9 March 2023). “*IRAK4*”, otherwise 51,135 (geneid), was chosen as the entry keyword. The process of filtering the retrieved variants was conducted, with only missense SNPs being selected for further analyses.

### 4.3. The Prediction of SNPs That Exhibit the Most Deleterious Implications

Six distinct computational tools were employed to predict the SNPs that have the most deleterious consequences on IRAK4 protein function. First, the SIFT tool (sorting intolerant from tolerant), which is available at (https://sift.bii.a-star.edu.sg; accessed on 20 March 2023), utilizes sequence homology along with the physical properties of amino acids to forecast the variants’ effects on protein function [84]. Moreover, the impact of amino acid substitutions on the structure and function of the investigated protein was evaluated by PolyPhen-2 (polymorphism phenotyping) through the use of physical along with comparative methodologies (http://genetics.bwh.harvard.edu/pph2; accessed on 24 March 2023) [85]. In addition, the identification of deleterious gene variants was accomplished through the SNPs&GO tool, which primarily relies on the functional annotation of the proteins under analysis (https://snps.biofold.org/snpsand-go/snps-and-go.html; accessed on 20 March 2023) [86]. The PHD-SNP tool employs support vector machines (SVMs) to anticipate the correlation between missense mutations and various human diseases (http://snps.biofold.org/phd-snp/phd-snp.html; accessed on 24 March 2023) [87]. The PANTHER tool, which stands for protein analysis through evolutionary relationships, employs evolutionary preservation calculations of amino acids to estimate the possibility of functional implications for non-synonymous variants [88]. The tool can be accessed at http://www.pantherdb.org/tools/csnpScoreForm.jsp; accessed on 24 March 2023. The SNAP2 tool was utilized, making use of its unique neural network capability to differentiate those effect variants from other neutral variants (https://rostlab.org/services/snap/; accessed on 20 March 2023) [89]. The SNPs that were identified as deleterious by all employed tools were deemed to be the most deleterious. Our objective was to enhance the precision and effectiveness of our analysis by integrating diverse tools, methodologies, and algorithms.

### 4.4. The Examination of the Impact of Genetic Variations on the Stability of IRAK4 Protein

The detrimental consequences of selected mutations on protein stability were investigated using I-Mutant 2.0 along with Mu-Pro tools. The I-Mutant 2.0 tool employs a support vector machine algorithm to anticipate the direction along with the magnitude of the change related to free energy (DDG), where negative values for DDG indicate a decreasing stability and positive values indicate an increasing stability (https://folding.biofold.org/i-mutant/i-mutant2.0.html; accessed on 25 March 2023) [90]. The I-Mutant 2.0 tool underwent testing on the ProTherm database, which contains extensive experimental data on alterations in free energy relevant to protein stability resulting from mutations [91]. The Mu-Pro tool utilizes a strong support vector machine methodology, which has demonstrated a precision rate of 84% during cross-validation along with verification procedures (http://mupro.proteomics.ics.uci.edu/; accessed on 25 March 2023) [92].

### 4.5. The Identification of the Positioning of SNPs on IRAK4 Protein Domains

The InterPro tool was employed to determine the locations of selected SNPs within IRAK4 protein domains, which can be accessed at https://www.ebi.ac.uk/interpro/; accessed on 25 March 2023. InterPro signifies a tool that is capable of conducting functional analysis of proteins, as well as detecting domains along with functional sites [93].

### 4.6. The Investigation of Secondary Structure

PSIPRED tool, available at http://bioinf.cs.ucl.ac.uk/psipred/; accessed on 26 March 2023, was employed to conduct an analysis of the secondary structure of the protein under investigation. This analysis was also utilized to identify the particular alignment of the modified amino acids within the investigated secondary structure. Additionally, an analysis was conducted on the secondary structures in the event of damaging mutations. The PSIPRED tool is capable of analyzing the secondary structure of a particular protein relying on position-specific matrices generated through PSI-BLAST [94].

### 4.7. The Examination of the Conservation of IRAK4 Protein Residues in Terms of Phylogenetics

Depending on ConSurf, the phylogenetic conservation regarding protein residues was examined. ConSurf is accessible online at https://consurf.tau.ac.il; accessed on 13 May 2023, and it has the capacity to conduct the necessary analysis by examining the phylogenetic relationships among diverse homologous sequences, by calculating the conversation score that ranges from grade 1 representing the most variable value to grade 9 representing the most conserved value [35,95,96].

### 4.8. Conducting an Analysis of the Impact of the Determined Variants on IRAK4 Protein Structure

The HOPE server (https://www3.cmbi.umcn.nl/hope/; accessed on 21 July 2023) was employed to analyze the implications of the most detrimental SNPs on the 3D structure of the IRAK4 protein. In addition to constructing homology models utilizing YASARA, HOPE relies on several sources to acquire relevant data and predict the implications of SNPs on the structure and function of the examined protein [97].

### 4.9. The Examination of Interactions between Genes

The production of a network that describes gene–gene interactions was accomplished through the utilization of GeneMANIA, which can be accessed at http://www.genemania.org; accessed on 13 May 2023. GeneMANIA has the capability of predicting genes that exhibit robust interactions based on diverse information and resources [98].

### 4.10. Assessing IRAK4 SNP Variants via Molecular-Docking-Coupled Dynamics Simulations

To evaluate the consequences of selected near-pocket SNPs on the druggability of IRAK4 proteins for inhibition, a molecular docking approach was conducted between the native/variant proteins and a reported potent small-molecule IRAK4 inhibitor, HG-12-6. The ternary structure of the native target protein with J84 was downloaded from RCSB Protein Data Bank (PDB ID: 6EGA; X-ray crystallographic atom resolution at 2.51 Å), serving as the control receptor [37]. Modeling of the missing loops was performed via SWISS-MODEL platform through the User-Template module (https://swissmodel.expasy.org/interactive#structure; accessed on 17 May 2023) using the PDB files (ID: 6ega for Holo and ID: 2oib for Apo) as templates and the IRAK4_HUMAN amino acid sequence deposited at Uniprot database (Entry: Q9NWZ3). Generated models with and without bound ligands were considered valid as per SWISS-MODEL MolProbity validation parameters, QMEANDisCo Global and QMEAN Z-Scores, as well as generated Ramachandran plots, which are one of the best quality indicators for experimental structure models [99] that can report the geometry and stereochemistry of the construct. Typically, the plots showed more than 85% of the residues being at favored zones, which is generally accepted for model data reliability [100,101] (Supplementary Materials; Figure S3).

Mutant models of IRAK4, both holo and apo, were built through the “Mutagenesis” module from PyMOL V.2.0.6. software and then subsequently energy-minimized by SWISS-PDBViewer, whereas the model target preparations proceeded using AutoDock/Vina as per the reported protocols [17,102]. Initial validation of the docking protocol was achieved via redocking the co-crystallized ligand while estimating the root-mean-squared deviation (RMSD Å) of the aligned co-crystallized and redocked poses [29,30,43,103]. Binding energies of the redocked and docked HG-12-6 within the canonical binding site (grid box; 70 × 70 × 70 Å along XYZ-cartesian coordinates) of native and SNP variant IRAK4 proteins, respectively, were estimated in terms of Kcal/mol values. The predicted ligand–target inhibition constant (*Ki*) was estimated relying on the obtained AutoDock/Vina binding energies (*Ki* = 10^binding energy ÷ 1.366^) [104]. Finally, the depicted 3D conformations of docked ligand–protein complexes, as well as residue-wise ligand interactions, were visualized via the PyMOL molecular graphics system [105].

Molecular dynamics simulations proceeded for the docked ligand–IRAK4 models using GROMACS-19 under CHARMM36m and CHARMM-General force fields [17,39,106,107]. The parameterization of all investigated ligands and generation of their respective topology files were automatically generated using the CHARMM-General force field program (Param-Chem project; https://cgenff.umaryland.edu/ accessed on 1 June 2023) [108]. Systems were set in the TIP3P-water model under periodic boundary conditions [109], having the protein target ionized at physiological pH = 7.4, while the system was neutralized using a sufficient number of chloride and potassium ions. System minimization was performed at the steepest-descent algorithm-minimization steps (5 ps; maximum energy tolerance of 1000 kJ/mol), then equilibrated at NVT (300 K) followed by NPT (300 K, 1 bar atm. pressure) ensembles for 500 ps each [109,110]. System production for 200 ns molecular dynamics simulations was set under the NPT ensemble (300 K, 1 bar atm. pressure) and Particle-Mesh/Ewald algorithm for computing far-ranged electrostatic interactions [111]. Far-range and short-range electrostatic interactions were set to a 1.0 nm cut-off. System production was set at 2 fs time step. Root-mean-squared deviations (RMSDs; Å), differences in RMS fluctuations (RMSFs; Å), and solvent-accessible surface area (SASA; Å^2^) were adopted regarding the whole trajectory analysis. Free binding energies for HG-12-6 at IRAK4-binding sites were calculated via the molecular mechanics/Poisson–Boltzmann (MM-PBSA; kJ/mol) single-trajectory calculation [112]. Conformational analysis and visualization of the simulated complexes at specified timeframes were performed using PyMOL software.

## 5. Conclusions

All in all, by analyzing *IRAK4* SNPs, the research detected ten SNPs as the most deleterious missense variants. Further analysis of stability aspects led to the nomination of certain nine deleterious SNPs. All these SNPS were positioned on a vital domain at highly conserved locations. Moreover, IRAK4 secondary structure alterations were associated with the L318S and L318F mutations. Detrimental impacts on the IRAK4 3D structure were also revealed with selected SNPs. Our molecular-docking-coupled dynamics analysis spotlighted potential mechanistic aspects where these identified SNPs could have hampered the inhibitor binding at the IRAK4 ATP-binding site. The loss of important ligand residue-wise contacts, as seen with L318F, the altered protein’s global flexibility at D311H, G195R, G198E, and L318S, the increased steric clashes with F330V, and even the electronic penalties by G198E and L318S towards the ligand–binding site interfaces were all suggested to be relevant for compromising the druggability profile of IRAK’s pocket against inhibition. The present analysis enables better monitoring and management of patients prone to various diseases relevant to IRAK4 malfunction and allows better understanding and improvement of IRAK4 inhibitor development.

## Figures and Tables

**Table 1:** The outcomes and evaluations of the damaging SNPs by all six distinct tools.

SNP	AA Change	SIFT		PolyPhen-2		SNP&GO			PHD-SNP		PANTHER		SNAP2		
		Prediction	Score	Prediction	Score	Prediction	Reliability Index (RI)	Probability	Prediction	Reliability Index (RI)	Prediction	Pdel	Prediction	Score	Expected Accuracy
rs1326024929	G195R	Deleterious	0	Probably damaging	1	Disease	8	0.89	Disease	8	Probably damaging	0.89	effect	95	95%
rs773245379	G198R	Deleterious	0	Probably damaging	1	Disease	8	0.901	Disease	8	Probably damaging	0.85	effect	94	95%
rs1592233207	G198E	Deleterious	0	Probably damaging	1	Disease	8	0.923	Disease	8	Probably damaging	0.85	effect	93	95%
rs149453390	K213M	Deleterious	0.01	Probably damaging	1	Disease	5	0.749	Disease	7	Probably damaging	0.89	effect	87	91%
rs1428184383	D311H	Deleterious	0	Probably damaging	1	Disease	5	0.754	Disease	4	Probably damaging	0.89	effect	92	95%
rs1265334986	L318S	Deleterious	0	Probably damaging	1	Disease	5	0.755	Disease	8	Probably damaging	0.89	effect	73	85%
rs1335719111	L318F	Deleterious	0	Probably damaging	1	Disease	3	0.645	Disease	6	Probably damaging	0.89	effect	50	75%
rs1941851625	F330V	Deleterious	0	Probably damaging	0.999	Disease	5	0.732	Disease	4	Probably damaging	0.89	effect	84	91%
rs774525787	R334W	Deleterious	0	Probably damaging	1	Disease	6	0.808	Disease	7	Probably damaging	0.89	effect	93	95%
rs748570560	R334Q	Deleterious	0.02	Probably damaging	0.999	Disease	4	0.703	Disease	2	Probably damaging	0.89	effect	85	91%

**Table 2:** Impact of genetic variations on IRAK4 protein stability.

SNP	AA Change	Mu-Pro		I-Mutant 2		
		Prediction	Delta Delta G	I-Mutant 2 Prediction	Reliability Index (RI)	DDG Value (kcal/mol)
rs1326024929	G195R	Decrease stability	−0.38	Decrease	1	−0.36
rs773245379	G198R	Decrease stability	−0.32	Decrease	2	−0.3
rs1592233207	G198E	Decrease stability	−0.22	Increase	4	0.52
rs149453390	K213M	Increase stability	0.26	Increase	4	0.76
rs1428184383	D311H	Decrease stability	−1.92	Decrease	3	−0.36
rs1265334986	L318S	Decrease stability	−1.65	Decrease	10	−2.29
rs1335719111	L318F	Decrease stability	−1.13	Decrease	9	−0.84
rs1941851625	F330V	Decrease stability	−1.32	Decrease	6	−2.17
rs774525787	R334W	Decrease stability	−0.78	Decrease	6	−0.71
rs748570560	R334Q	Decrease stability	−0.78	Decrease	7	−0.43

**Table 3:** Analysis of SNP positions on IRAK4 protein domains, secondary structure analysis, and phylogenetic conservation study.

SNP	AA Change	InterPro	PSIPRED		ConSurf			
		Location on Protein	Secondary Structure (Wild)	Secondary Structure (Mutated)	ConSurf Prediction	Conservation Score	Functional/Structural	Buried/Exposed
rs1326024929	G195R	Protein kinase domain	Coil	Coil	highly conserved	9	Functional	Exposed
rs773245379	G198R	Protein kinase domain	Strand	Strand	highly conserved	9	Structural	Buried
rs1592233207	G198E	Protein kinase domain	Strand	Strand	highly conserved	9	Structural	Buried
rs1428184383	D311H	Protein kinase domain	Coil	Coil	highly conserved	9	Functional	Exposed
rs1265334986	L318S	Protein kinase domain	Coil	Strand	highly conserved	9	Structural	Buried
rs1335719111	L318F	Protein kinase domain	Coil	Strand	highly conserved	9	Structural	Buried
rs1941851625	F330V	Protein kinase domain	Helix	Helix	highly conserved	9	Structural	Buried
rs774525787	R334W	Protein kinase domain	Helix	Helix	highly conserved	7		Exposed
rs748570560	R334Q	Protein kinase domain	Helix	Helix	highly conserved	7		Exposed

**Table 4:** The impact of the determined variants on IRAK4 protein structure.

SNP Id	AA Change	Amino Acid Properties	Location/Structure	Variants’ Impact on IRAK4 Protein
rs1326024929	G195R	The mutant residue differs from wild-type residue in size and charge. Being situated on the surface of our protein, the mutation can disrupt the needed interactions. Moreover, mutation could lead to the disruption of the protein’s local structure.	Residues situated near the mutated amino acid are annotated as a binding site, which could be affected by this mutation as the local structure could be impacted. In addition, the different amino acid properties could lead to disruption in the protein domain and its function.	Being situated in an important domain for protein activity that is also in contact with another important domain, this mutation could disrupt the needed interaction and impact protein function.
rs1592233207	G198E	The mutant residue differs from wild-type residue in charge and size. Being buried in a protein core, the mutant amino acid may not fit. Moreover, the mutation could lead to the disruption of the protein’s local structure.	Residues situated near the mutated amino acid are annotated as a binding site, which could be affected by this mutation as the local structure could be impacted. In addition, the different amino acid properties could lead to disruption in the protein domain and its function.	Being situated in an important domain for protein activity that is also in contact with another important domain, this mutation could disrupt the needed interaction and impact protein function.
rs773245379	G198R	The mutant residue differs from wild-type residue in size and charge. Being buried in a protein core, the mutant amino acid may not fit. Moreover, the mutation could lead to the disruption of the protein’s local structure.	Residues situated near the mutated amino acid are annotated as a binding site, which could be affected by this mutation as the local structure could be impacted. In addition, the different amino acid properties could lead to disruption in the protein domain and its function.	Being situated in an important domain for protein activity that is also in contact with another important domain, this mutation could disrupt the needed interaction and impact protein function.
rs1428184383	D311H	The mutant residue differs from wild-type residue in size and charge. Being buried in a protein core, the mutant amino acid may not fit.	Being situated in an active site, the mutation will result in disabling the protein function. In addition, the mutant amino acid leads to problems in making hydrogen bonds and salt bridges formed by wild residue. In addition, the different amino acid properties could lead to disruption in the protein domain and its function.	Being situated in an important domain for protein activity that is also in contact with another important domain, this mutation could disrupt the needed interaction and impact protein function.
rs1265334986	L318S	The mutant residue differs from wild-type residue in size, which could possibly result in losing external interactions. Moreover, there is a difference in hydrophobicity between wild residue and mutated one.	The 3D structure displayed the presence of interactions between wild-type amino acids and certain ligands, which could be lost in case of the mutated residue leading to disruption of protein function. In addition, the different amino acid properties could lead to disruption in the protein domain and its function.	Being situated in an important domain for protein activity that is also in contact with another important domain, this mutation could disrupt the needed interaction and impact protein function.
rs1335719111	L318F	The mutant residue differs from wild-type residue in size. Being situated on the surface of our protein, the mutation can disrupt the needed interactions.	The 3D structure displayed the presence of interactions between wild-type amino acids and certain ligands, which could be lost in case of the mutated residue leading to disruption of protein function. In addition, the different amino acid properties could lead to disruption in the protein domain and its function.	Being situated in an important domain for protein activity that is also in contact with another important domain, this mutation could disrupt the needed interaction and impact protein function.
rs1941851625	F330V	The mutant amino acid possesses a smaller size, which causes the presence of empty space in the protein core.	Residues situated near the mutated amino acid are annotated as a binding site, which could be affected by this mutation as the local structure could be impacted. In addition, the different amino acid properties could lead to disruption in the protein domain and its function.	Being situated in an important domain for protein activity that is also in contact with another important domain, this mutation could disrupt the needed interaction and impact protein function.
rs774525787	R334W	The mutant residue differs from wild-type residue in size and charge. Being situated on the surface of our protein, the mutation can disrupt the needed interactions. Moreover, there is a difference in hydrophobicity between wild residue and mutated one.	The different amino acid properties could lead to disruption in domain function.	Being situated in an important domain for protein activity that is also in contact with another important domain, this mutation could disrupt the needed interaction and impact protein function.
rs748570560	R334Q	The mutant residue differs from wild-type residue in charge and size with possible damage to interactions.	The different amino acid properties could lead to disruption in the protein domain and its function.	Being situated in an important domain for protein activity that is also in contact with another important domain, this mutation could disrupt the needed interaction and impact protein function.

**Table 5:** Docking parameters and binding affinities of HG-12-6 at the ATP-binding sites of the native and predicted IRAK4 SNP variant models.

IRAK4 Isoforms	Binding Energy (Kcal/mol)	RMSD * (Å)	*K*i (nM)	H-Bond Interactions [Binding Residues; Length (Å); Angle (°)]	Hydrophobic Interactions	π-Driven Interactions
D311H	−8.040	1.658	1300.534	Tyr262 sidechain (2.1 Å; 130.7°) Met265 mainchain (2.6 Å; 160.6°) Asp329 sidechain (1.9 Å; 150.0°) Asp329 mainchain (2.4 Å; 142.4°)	Ala211, Val236, Leu245, Val246, Val263, Tyr264, Gly268, Leu302, His309, His311, Leu318, Ile327	Tyr262 (3.8 Å) Phe330 (4.3 Å)
F330V	−8.986	1.557	264.018	Met265 mainchain (2.2 Å; 157.6°) Met265 mainchain (2.8 Å; 147.4°) Ile308 mainchain (1.9 Å; 144.6°) Asp329 sidechain (1.9 Å; 118.2°)	Phe197, Val200, Ala211, Val246, Val263, Gly268, His309, Leu318, Val330	Tyr262 (4.2 Å)
G195R	−9.007	1.174	255.044	Tyr262 sidechain (2.4 Å; 119.3°) Met265 mainchain (2.2 Å; 158.7°) Met265 mainchain (2.5 Å; 169.1°) Ile308 mainchain (2.2 Å; 131.9°) Asp329 sidechain (1.9 Å; 154.1°)	Val200, Ala211, Val236, Val246, Val263, Tyr264, Gly268, Ile308, His309, Leu318	Tyr262 (3.8 Å) Phe330 (5.0 Å)
G198E	−7.667	1.987	2439.170	Tyr262 sidechain (3.2 Å; 124.1°) Tyr264 sidechain (2.8 Å; 126.1°) Ile308 mainchain (2.1 Å; 133.9°) Asp329 sidechain (2.0 Å; 156.2°)	Val200, Ala211, Val236, Leu245, Val263, Gly268, Leu302, His309, Leu318	Tyr262 (4.1 Å) Phe330 (5.0 Å)
G198R	−9.145	1.062	202.072	Glu233 sidechain (2.5 Å; 111.2°) Met265 mainchain (2.0 Å; 151.6°) Met265 mainchain (2.1 Å; 156.6°) Ile308 mainchain (1.8 Å; 140.4°) Asp329 sidechain (2.4 Å; 160.1°)	Phe197, Val200, Ala211, Val246, Val263, Tyr264, Gly268, His309, Leu318	Phe197 (4.4 Å) Tyr262 (4.0 Å) Phe330 (4.9 Å)
L318S	−9.162	1.145	196.292	Glu233 sidechain (2.4 Å; 151.8°) Met265 mainchain (2.1 Å; 154.1°) Met265 mainchain (2.1 Å; 163.3°) Ile308 mainchain (3.5 Å; 126.5°) Asp329 sidechain (2.0 Å; 166.7°)	Ala211, Val236, Val246, Val263, Gly268, Leu302, His307, Ile308, His309	Tyr262 (4.0 Å) Phe330 (5.0 Å)
L318F	−8.313	1.633	821.136	Met265 mainchain (1.9 Å; 162.3°) Met265 mainchain (2.0 Å; 156.3°) Ile308 mainchain (2.0 Å; 131.0°) Asp329 sidechain (1.9 Å; 160.2°)	Val200, Ala211, Val236, Val246, Val263, Tyr264, Gly268, Leu302, Ile308, His309, Phe318	Tyr262 (4.1 Å) Phe330 (4.4 Å)
Native	−9.333	0.947	147.014	Tyr262 sidechain (2.7 Å; 124.6°) Met265 mainchain (2.0 Å; 157.3°) Met265 mainchain (2.1 Å; 130.4°) Ile308 mainchain (2.3 Å; 124.1°) His309 mainchain (3.3 Å; 128.6°) Asp329 sidechain (1.7 Å; 157.0°)	Ala211, Val236, Val246, Val263, Tyr264, Gly268, Leu302, His309, Leu318, Ile327	Phe330 (4.9 Å) Tyr262 (3.9 Å)

* Root-mean-squared deviation of redocked/docked HG-12-6 at native and SNP variants of IRAK4 target protein in relation to the co-crystallized ligand.

## Data Availability

The original contributions presented in the study. Further inquiries can be directed to the corresponding authors.

## Supplementary Materials

The following supporting information can be downloaded at https://www.mdpi.com/article/10.3390/jpm13121648/s1, Figure S1. Investigation of IRAK4’s secondary structure produced by PSIPRED server: 2A) secondary structure of the wild form, 2B) secondary structure with L318S mutation, and 2C) secondary structure with L318F mutation; Figure S2. Investigation of IRAK4’s phylogenetic conservation produced by ConSurf server; Figure S3. Overall structural quality assessment of IRAK4 Holo (A) and Apo (B) modelled missing loops at the respective deposited PDF.file, using SWISS-MODEL MolProbity validation parameters; Table S1: Monitored ΔRMSF for HG-12-6-bound IRAK4 protein across the whole molecular dynamics runs.
