# Supplementary material for: Unraveling Extremely Damaging IRAK4 Variants and Their Potential Implications for IRAK4 Inhibitor Efficacy
Source: J Pers Med. 2023 Nov 26;13(12):1648. doi: 10.3390/jpm13121648 (PMC10744719; doi:10.3390/jpm13121648)
Supplement: Supplementary file 1 [file jpm-13-01648-s001.zip › jpm-2670435-supplementary.pdf]

Article

Unraveling IRAK4 extremely damaging variants and their potential implications on IRAK4 inhibitors efficacy

Mohammed Y Behairy [1], Refaat A. Eid [2], Hassan M. Otifi [2], Heitham Mohammed[3], Mohammed A. Alshehri [4], Ashwag Asiri[4], Majed Aldehri [3], Mohamed Samir A. Zaki[3, 5], Khaled M. Darwish[6], Sameh S. Elhady[7], Nahla H. El-shaer[8], Muhammad Alaa Eldeen[9]*

[1] Department of Microbiology and Immunology, Faculty of Pharmacy, University of Sadat City, Sadat City, Menoufia, Egypt; mohamedyehya950@gmail.com

[2] Department of Pathology, College of Medicine, King Khalid University, P.O. 61421, Abha, Saudi Arabia (raeid@kku.edu.sa), (hotifi@kku.edu.sa).

[3] Department of Anatomy, College of Medicine, King Khalid University, P.O. 61421, Abha, Saudi Arabia. (mutwakilheitham@gmail.com), (maldehri@kku.edu.sa), (mszaki@kku.edu.sa),

[4] Department of Child Health, College of Medicine, King Khalid University, P.O. Box 62529, Abha, Saudi Arabia. (mohamed8964@hotmail.com), (asalasiri@kku.edu.sa)

[5] Department of Histology and Cell Biology, College of Medicine, Zagazig University, Zagazig, Egypt

[6] Department of Medicinal Chemistry, Faculty of Pharmacy, Suez Canal University, Ismailia 41522, Egypt; khaled_darwish@pharm.suez.edu.eg

[7] Department of Natural Products, Faculty of Pharmacy, King Abdulaziz University, Jeddah 21589, Saudi Arabia; ssahmed@kau.edu.sa

[8] Zoology Department, Faculty of Science, Zagazig University, Zagazig 44511, Egypt; nhelshaer@zu.edu.eg

[9] Cell Biology, Histology & Genetics Division, Zoology Department, Faculty of Science, Zagazig University, Zagazig 44519, Egypt; dr.muhammadalaa@gmail.com

* Correspondence: dr.muhammadalaa@gmail.com

Supporting materials

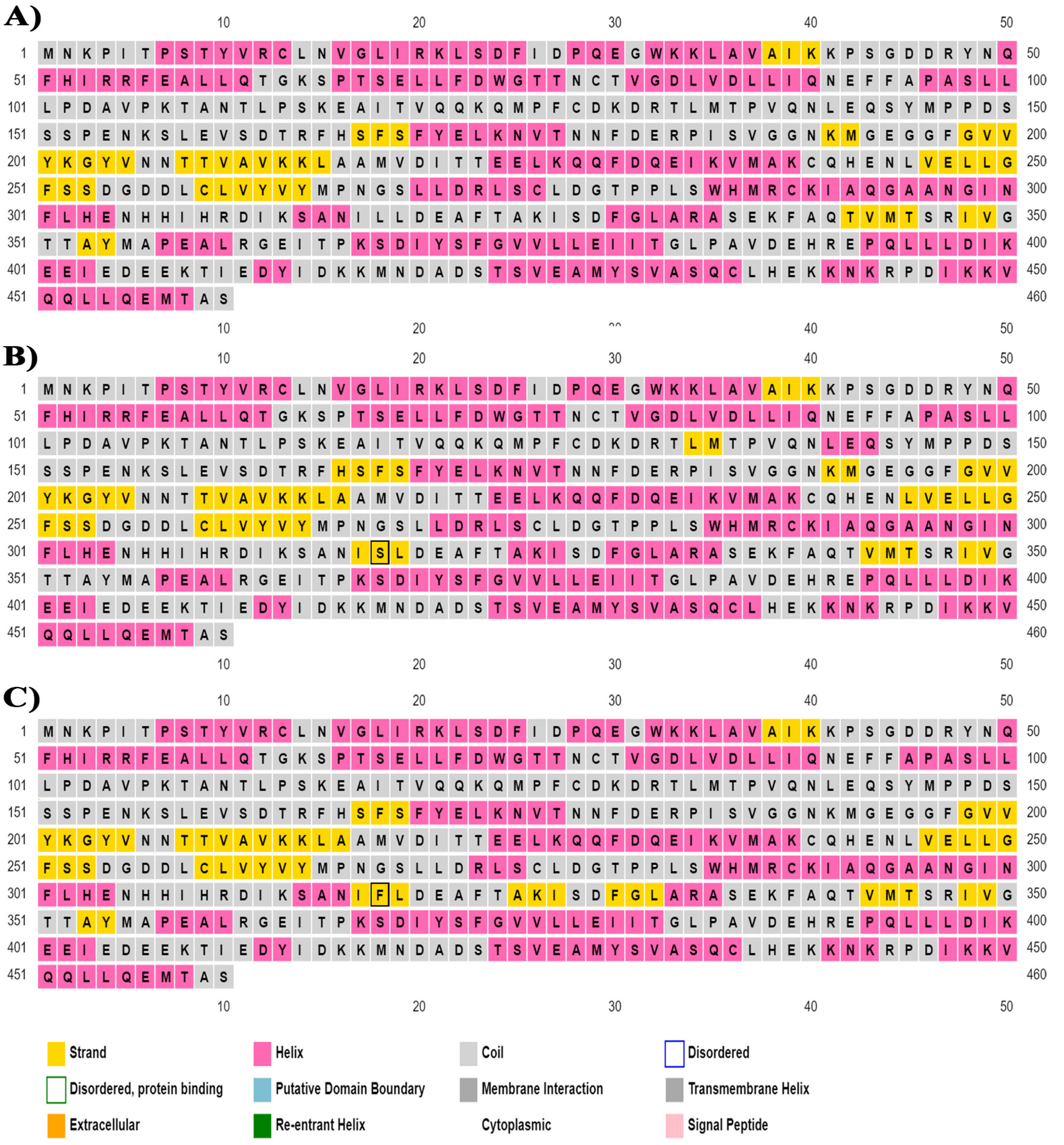

Figure S1. Investigation of IRAK4's secondary structure produced by PSIPRED server: (A) secondary structure of the wild form, (B) secondary structure with L318S mutation, and (C) secondary structure with L318F mutation.

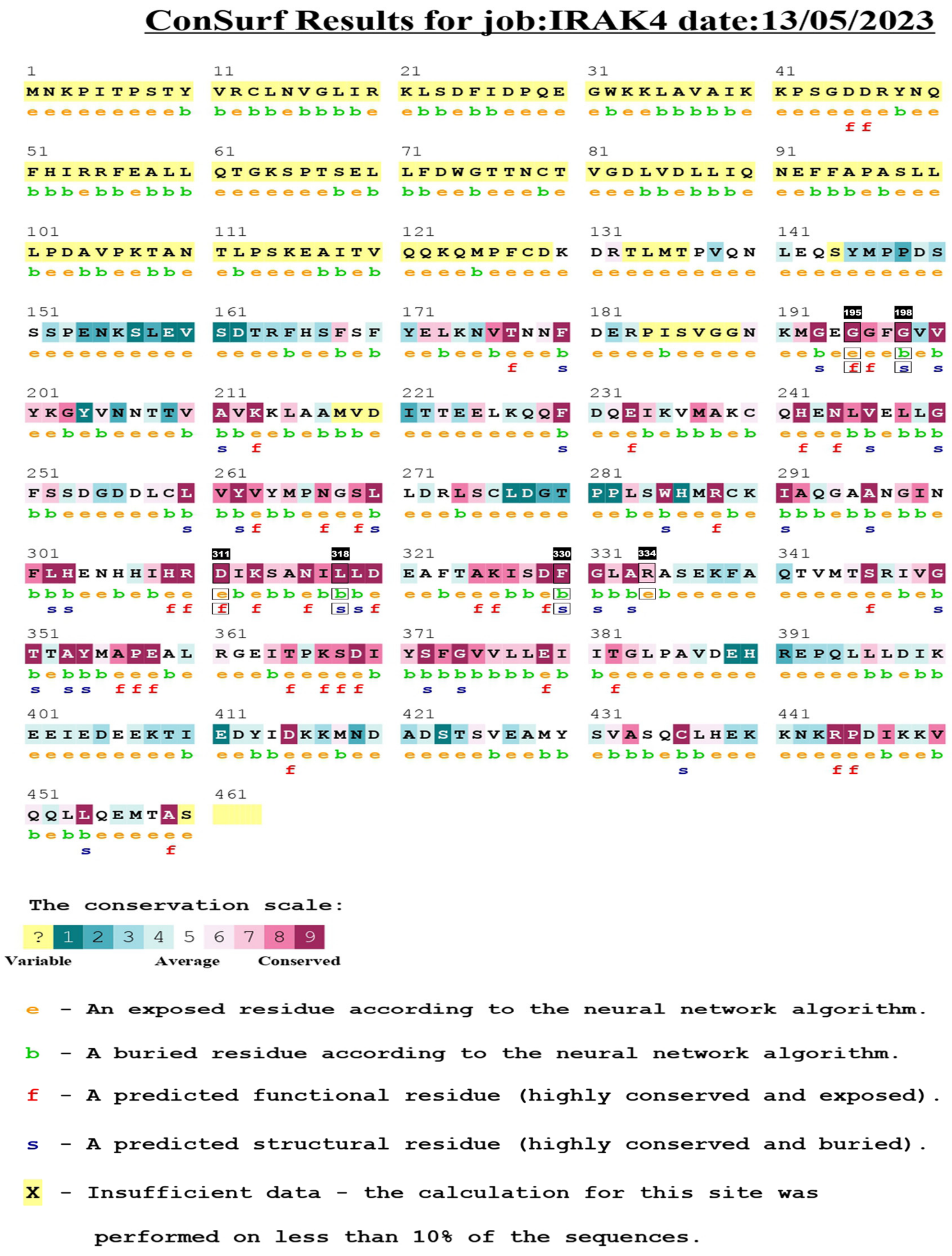

Figure S2. Investigation of IRAK4's phylogenetic conservation produced by ConSurf.

(A)

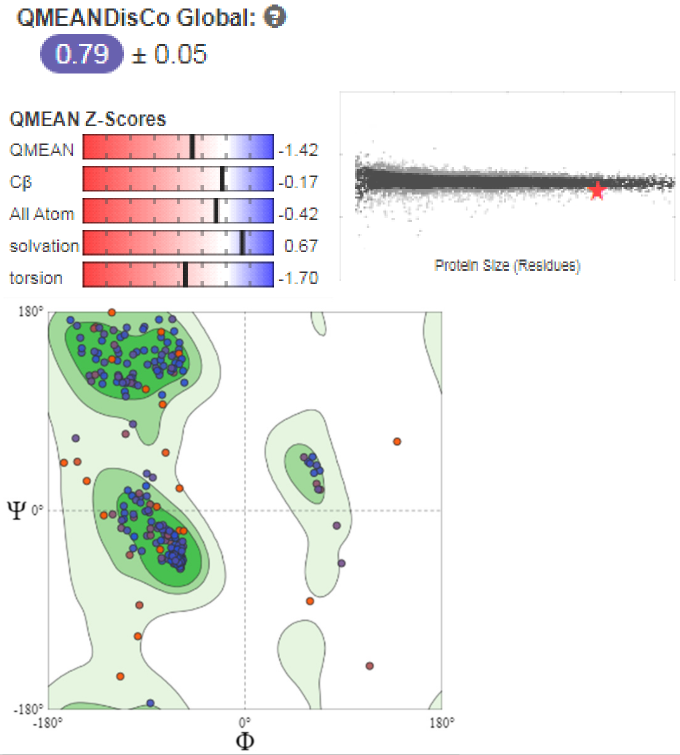

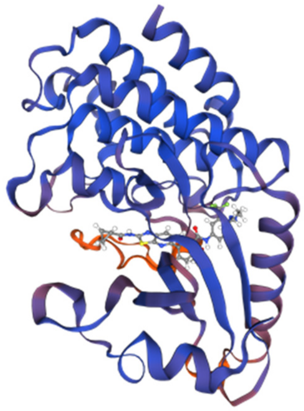

MolProbity Score = 2.23
Clash Score = 8.20
Ramachandran Favoured = 92.81%
Ramachandran Outliers = 2.05%
Rotamer Outliers = 2.75%

(B)

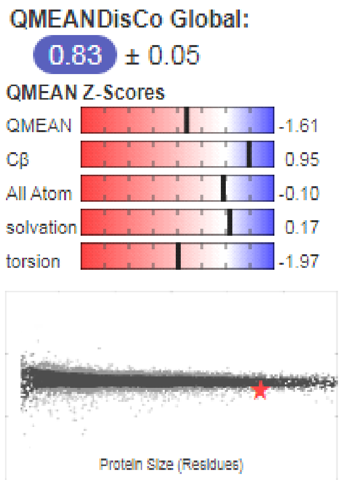

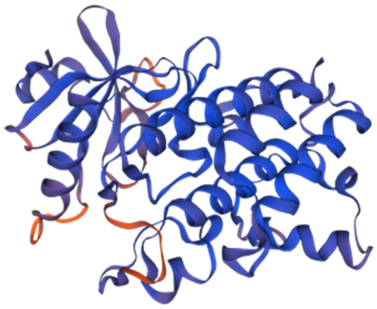

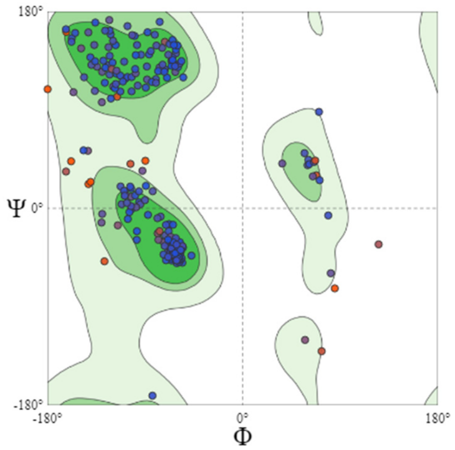

MolProbity Score = 1.18
Clash Score = 0.22
Ramachandran Favoured = 92.86%
Ramachandran Outliers = 2.38%
Rotamer Outliers = 1.56%

Figure S3. Overall structural quality assessment of IRAK4 Holo (**A**) and Apo (**B**) modelled missing loops at the respective deposited PDF.file, using SWISS-MODEL MolProbity validation parameters

Table S1. Monitored ΔRMSF for HG-12-6 bounded IRAK4 protein across the whole molecular dynamics runs

IRAK4 important structural domains	Residues	D311H	F330V	G195R	G198E	G198R	L318S	L318F	Native
	**Pro184**	-0.83	-0.85	-0.16	-0.61	-0.12	-0.62	-0.22	-0.10
	**Ile185**	-0.09	-0.16	**0.44**	**0.03**	**0.46**	-0.06	**0.31**	**0.49**
***N*-lobe**	**Ser186**	**0.92**	**0.18**	**0.73**	**0.15**	**1.17**	**0.78**	**1.12**	**0.83**
**Schellman loop**	**Val187**	**1.46**	**1.20**	**1.62**	**1.05**	**1.78**	**1.19**	**1.64**	**1.80**
	**Gly188**	**1.76**	**2.03**	**2.34**	**1.83**	**2.32**	**1.64**	**2.10**	**2.56**
	**Gly189**	**1.63**	**2.02**	**2.42**	**2.13**	**2.07**	**1.49**	**1.94**	**2.63**
	**Gly193**	-0.58	-1.42	-0.88	-1.19	-0.28	-0.59	-0.14	-0.73
	**Glu194**	-0.90	-1.00	-0.35	-1.21	-0.46	-1.50	-0.57	-0.71
***N*-lobe**	**Gly/x195**	0.22	**0.23**	**-0.25**	**0.13**	0.24	0.85	**0.28**	0.95
**G-rich loop**	**Gly196**	-0.14	**0.70**	**1.26**	**-0.36**	0.08	-0.40	**0.64**	**0.54**
	**Phe197**	**0.63**	**1.50**	**1.96**	**0.27**	**0.33**	**0.41**	**1.43**	**1.53**
	**Gly/x198**	**1.30**	**1.07**	**0.45**	**0.22**	**0.87**	1.19	**0.95**	**1.39**
	**ILle221**	-1.58	-2.13	-0.04	-2.57	-1.82	-1.46	**0.49**	**0.59**
	**Thr222**	0.24	-2.50	**1.45**	-0.16	-0.88	0.28	**1.50**	**1.86**
	**Thr223**	**0.73**	-1.58	**2.00**	**0.36**	-1.36	**0.33**	**1.72**	**2.04**
	**Glu224**	**0.52**	-1.19	**1.32**	**0.47**	-0.67	0.06	**1.37**	**1.27**
	**Glu225**	**0.70**	**0.89**	**1.47**	**0.45**	-1.11	-0.08	**1.26**	**1.30**
	**Leu226**	-0.17	**0.59**	**0.94**	-0.54	-0.95	-0.90	**0.49**	**0.77**
	**Lys227**	0.27	**0.88**	**1.11**	0.26	**0.32**	-0.23	**0.94**	**1.03**
***N*-lobe**	**Gln228**	-0.70	-0.44	-0.31	-1.16	-2.03	-1.36	-0.34	-0.50
**αC-helix**	**Gln229**	-0.93	-0.44	-0.26	-1.77	-2.41	-1.71	-0.58	-0.56
	**Phe230**	-0.85	-0.10	0.03	-1.16	-1.26	-1.44	-0.36	-0.14
	**Asp231**	-0.50	-0.28	-0.20	-0.82	-0.56	-1.04	-0.20	-0.32
	**Gln232**	-0.62	-0.46	-0.39	-1.75	-0.77	-1.35	-0.49	-0.65
	**Glu233**	-0.78	-0.18	-0.08	-1.57	-0.63	-1.39	-0.45	-0.35
	**Ile234**	-0.57	-0.17	-0.05	-0.63	-0.42	-1.04	-0.26	-0.22
	**Lys235**	-0.47	-0.49	-0.37	-1.33	-0.33	-1.01	-0.33	-0.65
	**Val236**	-0.79	-0.54	-0.38	-1.70	-0.44	-1.42	-0.48	-0.74

	**Met237**	-0.83	-0.39	-0.08	-0.69	-0.55	-1.32	-0.36	-0.37
	**Ala238**	-0.68	-0.77	-0.36	-0.99	-0.48	-0.98	-0.34	-0.79
	**Lys239**	-0.89	-1.10	-0.63	-1.78	-1.17	-1.56	-0.51	-1.39
	**Cys240**	-1.12	-1.02	-0.47	-1.27	-1.78	-1.87	-0.41	-1.11
**Hinge region**	**Tyr262 (GK)**	-0.14	-0.33	-0.12	-0.35	-0.18	-0.20	-0.08	-0.01
	**Val263**	-0.17	-0.50	-0.31	-0.48	-0.21	-0.23	-0.14	-0.13
	**Tyr264**	-0.32	-0.64	-0.38	-0.64	-0.29	-0.42	-0.25	-0.18
	**Met265**	-0.23	-0.23	-0.20	-0.55	-0.39	-0.28	-0.11	-0.11
	**Pro266**	-0.40	-0.29	-0.55	-0.83	-0.32	-0.21	-0.23	-0.05
	**Asn267**	-0.59	-0.20	-0.15	-0.53	-0.59	-0.42	-0.17	-0.15
	**Gly268**	-0.27	-0.13	0.06	-0.42	-0.13	-0.14	0.08	-0.01
	**Ser269**	-0.41	-0.21	-0.04	-0.40	-0.25	-0.27	-0.06	-0.10
	**Leu270**	-0.25	-0.03	0.11	-0.18	-0.05	-0.11	0.01	**0.35**
**α-DE loop**	**Cys276**	-0.41	-0.38	-0.26	-0.30	-0.38	-0.26	-0.36	-0.267
	**Leu277**	-0.27	-0.37	-0.22	-0.29	-0.21	-0.11	-0.38	-0.044
	**Asp278**	-0.44	-0.59	-0.40	-0.55	-0.34	-0.27	-0.58	-0.18
	**Gly279**	-0.59	-0.53	-0.35	-0.46	-0.50	-0.44	-0.50	-0.275
	**Thr280**	-0.35	-0.03	0.12	**0.00**	-0.28	-0.21	-0.07	**0.04**
	**Pro281**	-0.02	-0.08	-0.15	-0.09	-0.03	-0.10	-0.22	**0.19**
	**Pro282**	-0.26	-0.11	**0.00**	-0.05	-0.21	-0.12	-0.06	**0.05**
**Leu318 with vicinal residues**	**Asn316**	-0.25	-0.35	-0.30	-0.34	-0.28	-0.41	-0.60	-0.34
	**Ile317**	-0.20	-0.06	0.00	-0.33	-0.30	-0.38	-0.40	0.00
	**Leu/x318**	-0.09	-0.12	0.03	-0.09	-0.04	-0.22	-0.49	0.00
	**Leu319**	-0.24	-0.17	-0.09	-0.11	-0.12	-0.39	-0.56	-0.08
	**Ap320**	-0.25	-0.27	-0.24	-0.11	-0.13	-0.38	-0.79	-0.21
**Activation Segment**	**Asp329**	-0.41	-0.27	-0.31	-1.19	-0.17	-0.24	-0.24	-0.09
	**Phe/x330**	-0.51	-0.98	-0.58	-1.33	-0.44	-0.42	-0.34	-0.261
	**Gly331**	-0.78	-0.57	-0.71	-2.37	-0.89	-0.94	-0.56	-0.545
	**Leu332**	-0.93	-1.79	-0.94	-2.35	-1.10	-0.86	-1.24	-0.63
	**Ala333**	-1.01	-2.13	-1.15	-3.11	-1.07	-0.81	-1.12	-0.67
	**Arg334**	-1.34	-2.01	-1.90	-4.32	-1.45	-1.19	-2.85	-0.56
	**Ala335**	-1.77	-3.84	-4.14	-5.33	-1.67	-2.29	-2.45	-0.55
	**Ser336**	-2.93	-3.61	-4.32	-5.18	-1.62	-3.05	-2.63	-0.48
	**Glu337**	-3.57	-3.50	-4.19	-5.62	-2.08	-4.38	-2.52	-0.64
	**Lys338**	-4.36	-4.42	-5.32	-5.82	-2.04	-5.30	-2.79	-0.93
	**Phe339**	-2.95	-3.77	-4.19	-4.38	-1.05	-4.75	-2.30	0.13
	**Ala340**	-1.36	-3.20	-2.57	-4.88	-0.58	-2.15	0.09	**0.84**
	**Gln341**	-1.47	-1.18	-2.78	-5.92	-2.33	-1.45	0.17	**0.89**
	**Thr342**	-1.92	-1.54	-2.92	-6.08	-4.71	-1.75	-0.69	**0.45**
	**Val343**	-2.35	-2.10	-1.91	-6.08	-4.41	-0.86	-0.40	0.14
	**Met344**	-2.13	-2.40	-2.07	-5.88	-4.65	0.04	-0.44	-0.18
	**Thr345**	-4.07	-3.77	-3.00	-6.82	-5.01	-0.55	-1.84	-0.70
	**Ser346**	-4.53	-3.88	-2.49	-5.73	-3.92	-1.34	-2.40	-0.81
	**Arg347**	-3.91	-4.06	-1.47	-4.50	-3.72	-0.52	-2.82	-0.74
	**Ile348**	-4.31	-3.99	-1.03	-3.30	-2.53	0.16	-1.82	-0.34
	**Val349**	-5.09	-1.67	0.13	-1.86	-1.51	-0.28	-0.32	-0.05
	**Gly350**	-3.70	-1.92	**0.54**	-1.00	-0.43	-0.59	-0.73	**0.47**

**Thr351**	-1.93	-0.15	**1.17**	**0.37**	-1.20	**0.43**	-0.36	**1.09**
**Thr352**	0.00	**1.76**	**1.92**	**1.26**	**0.91**	**1.05**	**0.67**	**1.34**
**Ala353**	-0.29	**0.76**	**1.10**	**0.42**	**0.74**	0.16	**0.63**	0.16
**Tyr354**	-0.45	**0.08**	0.07	-0.31	**0.12**	-0.18	-0.18	-0.36
**Met355**	-0.12	-0.07	-0.41	-0.65	0.14	0.02	0.13	-0.28
**Ala356**	-0.61	-0.26	-0.68	-0.79	-0.19	-0.38	-0.25	-0.40
**Pro357**	-0.49	-0.43	-1.00	-1.02	-0.20	-0.40	-0.21	-0.58
**Glu358**	-0.73	-0.49	-1.28	-1.14	-0.50	-0.72	-0.51	-0.74

[a] *ΔRMSF ≥ 0.30 Å cut-off are in bold red numbers inferring residues showing significant immobility/inflexibility.*
